# Synthesis, structural elucidation, and molecular docking of diclofenac-derived hydrazone metal complexes with anti-inflammatory and anticancer potential

**DOI:** 10.1038/s41598-025-27143-1

**Published:** 2025-11-26

**Authors:** A. M. Abbas, H. A. Salem, A. S. Orabi

**Affiliations:** Faculty of Science, Chemistry Department, Suez Canal University, Ismailia, 41522 Egypt

**Keywords:** Diclofenac-derived hydrazone, Metal complexes, Anti-inflammatory, Anticancer, In silico, In vitro, Biochemistry, Cancer, Chemical biology, Chemistry, Computational biology and bioinformatics, Drug discovery

## Abstract

A novel Schiff base ligand, (E)-2-(2-((2,6-dichlorophenyl)amino)phenyl)-N’-(2-hydroxynaphthalen-1-yl)methylene)acetohydrazide, was synthesized and complexed with Cu(II), Ni(II), Co(II), Gd(III), La(III), and Ag(I) ions. The resulting metal complexes were characterized using FTIR, UV–Vis, 1H NMR, ESR, XRD, thermal analysis, and magnetic susceptibility measurements. Spectroscopic and magnetic data confirmed octahedral geometries for Co(II), Ni(II), and Cu(II), square antiprismatic and tricapped trigonal prismatic geometries for Gd(III) and La(III), respectively, and a tetrahedral geometry for Ag(I). The anti-inflammatory activity was evaluated using ELISA-based COX-1/COX-2 inhibition assays. The HDN ligand exhibited potent COX-2 inhibition (IC₅₀ = 0.06 µM) with a selectivity index (SI) of 174.5, outperforming diclofenac sodium (SI = 4.52) and indomethacin (SI = 1.25), and approaching rofecoxib (SI = 725). The Cu(II) and Gd(III) complexes showed strong cytotoxicity in MTT assays against MCF-7 (IC₅₀ = 0.65 and 0.80 µM) and HepG-2 (IC₅₀ = 1.00 and 2.47 µM) cell lines, significantly surpassing the HDN ligand and standard drugs such as 5-fluorouracil (IC₅₀ = 3.95 µM, MCF-7) and cisplatin (IC₅₀ = 15.24 µM, MCF-7). In silico studies including molecular docking and ADME profiling supported the experimental findings. The HDN ligand and Cu(II) complex exhibited strong binding affinities to COX-2 (− 22.87 kcal/mol), HepG-2 (− 32.56 and − 31.76 kcal/mol), and MCF-7 (− 25.29 and − 20.20 kcal/mol) receptors. SwissADME and BOILED-Egg models predicted high gastrointestinal absorption for the HDN ligand and favorable pharmacokinetic profiles for the metal complexes. The present results provide a preclinical proof-of-concept for diclofenac-derived Schiff base metal complexes as dual anti-inflammatory and anticancer agents. However, their therapeutic potential remains to be validated through stability, mechanistic, and in vivo investigations.

**Supplementary Information:**

The online version contains supplementary material available at 10.1038/s41598-025-27143-1.

## Introduction

Inflammation is a critical reaction to tissue damage caused by various factors, such as physical trauma and exposure to chemicals or biological agents. Both short-term (acute) and long-lasting (chronic) inflammation are vital processes in restoring the body’s equilibrium^1^. Activating leukocytes in combating pathogens and tumor cells and clearing tissue debris during inflammation can generate oxidants and cytotoxic cytokines^2,3^. Although these mechanisms are typically well-regulated, there are occasions when they can become dysregulated, leading to an extended inflammatory response. This can result in a self-perpetuating state that exacerbates tissue damage^4^. The inflammatory process starts with an immediate reaction upon the finding of antigens or damage, prompting the recruitment of neutrophils to the affected area through the activity of chemoattractant mediators. Afterward, monocytes/macrophages infiltrate and become the dominant type of immune cells present within 48 h^5,6^.

Neutrophil activation prompts the release of cytokines, which enhance the inflammatory reaction, resulting in lymphocyte infiltration, vascular changes, swelling, and tissue degradation by enzymes. Granulocytes move into inflamed regions and stimulate the production of arachidonic acid metabolites and leukotrienes through the action of lipoxins^7^.

There are about 50 diverse medications classified as non-steroidal anti-inflammatory drugs (NSAIDs) commonly used to alleviate pain. They demonstrate varying anti-inflammatory, antipyretic, and analgesic effects^8^. Nevertheless, many NSAIDs share a similar range of side effects, especially concerning gastrointestinal issues. Consequently, there is ongoing interest in developing new drugs to address chronic inflammation and pain while minimizing these complications^9^. Indeed, the majority of NSAIDs are known for their significant gastrointestinal (GI) toxicity, which can be life-threatening. The gastrointestinal (GI) toxicity linked to all traditional NSAIDs is caused by the presence of the free carboxylic acid (-COOH) group^10^.

Metal complexes are important innovations in medicinal chemistry, demonstrating significant potential in enhancing drug efficacy. These complexes can increase drug bioavailability and selectivity and reduce toxicity. Specifically, metal complexes formed with non-steroidal anti-inflammatory drugs (NSAIDs) have shown notable improvements in anti-inflammatory and analgesic activities, allowing for better drug-target interaction and improved therapeutic outcomes while minimizing side effects such as gastrointestinal issues commonly associated with these drugs. For instance, the copper-aspirin complex has demonstrated more excellent selectivity in inhibiting the COX-2 enzyme than aspirin alone, thereby reducing the risk of gastric ulcers^11^. Additionally, the zinc-aceclofenac complex has proven to be more stable in acidic conditions, reducing the risk of gastric ulcers while maintaining its anti-inflammatory efficacy^12^. Thus, the strategic design of metal complexes offers promising approaches to improving drug formulations, leading to more effective treatments with fewer adverse effects.

Recent reports on Schiff base metal complexes further support the biomedical significance of this class of compounds^13–19^.

Molecular docking is a computational technique that anticipates the formation of a stable intermolecular complex when at least two particles interact and bind together. A theoretical method demonstrates how a compound binds to DNA via non-covalent interactions. This theoretical process is the most suitable for structure-based drug design^20,21^. Molecular docking is significant in determining the compound to continue progressing to experimental studies. Data gathered from theoretical and experimental studies can indicate a compound’s potential as a drug candidate^22^.

The research unveils a newly synthesized Schiff base compound and its metal complexes, representing a novel discovery. The synthesized compounds underwent characterization employing various techniques, including mass spectroscopy, ^1^HNMR, elemental analysis, conductivity measurements, magnetic properties assessment, ESR, X-ray diffraction, and spectral analysis using FTIR and UV–Vis. Thermal methods such as TG and DTG were also utilized for further characterization. The anti-inflammatory activity of certain compounds was assessed in vitro using Enzyme-linked immunosorbent assay (ELISA). The prepared compounds were assessed for their capacity to inhibit cell proliferation in HepG-2 and MCF-7 cancer cell lines using the MTT-Cytotoxicity assay protocol.

## Experimental

### Materials

All the chemicals utilized in the current investigation were sourced from Sigma-Aldrich and were pure grade, requiring no additional purification before use.

### Napthaldehyde hydrazone

Diclofenac acid, diclofenac ethyl ester, and diclofenac hydrazide were synthesized following previously described procedures^23–25^. A diclofenac hydrazide methanolic solution (1.0 mmol, 0.310 g) was prepared at room temperature. 2-hydroxy-1-naphthaldehyde (1 mmol, 0.172 g) was added to this solution. The reaction mixture was then refluxed with continuous stirring for 2 h at ambient temperature. A yellow precipitate formed after the methanolic solvent volume was reduced by half. The resulting product was filtered, washed with methanol for purification, and dried under reduced pressure to remove any residual solvent. It was then stored in a desiccator containing anhydrous CaCl_2_ to ensure dryness.

### Synthesis of the target complexes

The intended metal complexes were synthesized using the ensuing method. The Schiff base (1.0 mmol, 0.464 g) was disbanded in 20 mL of acetone. The metal nitrate (or chloride) (0.5 mmol) was dispersed in 10 mL of acetone and added gradually to the ligand solution while stirring. The pH was adjusted to 7–7.7 using NH_4_OH for all complexes except for Gd(III) and La(III), which were adjusted to 4.5–5.5. Subsequently, the entire mixture was stirred and refluxed for 2–6 h. The precipitate underwent filtration, rinsing with hot acetone, and finally drying under vacuum with anhydrous CaCl_2_.

### In vitro* investigates*

#### ELISA

The compounds’ ability to inhibit COX-1 (ovine) and recombinant COX-2 (human, measured by IC_50_ values in µM) assessed using ELISA kit (Cayman Chemical, Ann Arbor, MI, USA. item No. 560131), following Cayman Chemical’s standardized methods^26,27^.

#### In vitro antitumor activity valuation (MTT– Cytotoxicity assay protocol)

MCF-7 and HepG-2 cell lines (provided by the Cell Culture Department, VACSERA, Egypt) were seeded in each 96-well tissue culture plate well at a concentration of 1 × 10^5^ cells/ml, adding 100 µl/well. Incubation at 37 °C followed for 24 h until a full monolayer was established. The growth medium was later aspirated, and the cell monolayer was rinsed twice using wash media. It was then subjected to a two-fold dilution in an RPMI medium containing 2% serum, which is regarded as a maintenance medium. Later, 0.1 ml of each dilution was added to individual wells. Three wells were reserved for use as controls and received only the maintenance medium. The plate was then incubated at 37 °C and further monitored accordingly. The marks of toxicity to be checked on the cells include disruption of the cell monolayer, cell rounding and shrinking, or the appearance of granules. MTT solution is prepared from BIO BASIC CANADA INC with a 5 mg/ml strength in PBS. To ensure the MTT and media are well mixed, add 20 µl of MTT solution to each well and shake the plate. The MTT will be metabolized by incubating the plate at 37 °C with 5% CO2 for one to five hr. If there is any residual substance, get rid of the rest by drying the plate on paper towels. Add DMSO (200 µl) to dissolve the formazan (MTT metabolic product) and then put the plate on the shaking table set at 150 rpm for 5 min to assure that the formazan is fully dissolved into the solvent. Measure the optical density at 560 nm and subtract the background signal at 620 nm. The optical density value reflects directly the number of cells^28–30^. MCF-7 (breast cancer) and HepG-2 (liver cancer) cell lines.

### In silico* physicochemical descriptors, pharmacokinetic properties, and bioactivity prediction*

The drug-likeness, pharmacokinetics, and physicochemical characteristics of compounds were assessed in silico utilizing the SwissADME web application^31^. Numerous factors were taken into account in the analysis, including the counts of heavy atoms, rotatable bonds, H-bond donors, and acceptors. The proportion of carbon bond saturation (C-sp3), which is the ratio of sp3 hybridized carbons to the overall number of carbons, was also calculated as part of the analysis. Moreover, it encompassed determining molar refractivity, topological polar surface area (TPSA), water solubility (S) expressed as LogS (Silicos-IT), and lipophilicity described by LogP, estimated using both the additive XLogP3 and the Wildman-Crippen method^32,33^. Among the pharmacokinetic properties estimated for each compound were P-glycoprotein substrates (Pgp substrates), gastrointestinal absorption (GI absorption), skin permeability (LogKp), and blood–brain barrier permeability (BBB permeability). Veber’s rule-based approach, which indicates that compounds with a polar surface area ≤ 140 Å2 and rotatable bonds equal ten or fewer are more likely to have appropriate bioavailability, was utilized to evaluate drug-likeness^34^.

Molecular docking models assessed the interactions of the synthesized diclofenac derivative metal complexes with the target proteins 4XO6, 5EQG, COX-1, and COX-2. The simulations were performed using AutoDock Vina (version 1.2.5) with a semi-flexible docking protocol, allowing ligand flexibility while keeping the receptor proteins rigid. The proteins’ 3D structures were obtained from the Protein Data Bank (PDB)^35^. To prepare the protein models, hydrogen atoms were included, water molecules were eliminated, and only essential heteroatoms at the active sites were retained.

Ligand molecules were optimized in MarvinSketch to create their 3D shapes and later persuaded into PDBQT format using AutoDock Tools. Gasteiger charges were allocated, and non-polar hydrogens were joined. A grid box was then applied to define the docking region, ensuring coverage of active site residues linked to NSAID interactions. The grid was focused on the dynamic position, providing sufficient universe to explore various binding conformations for the ligands. The poses with the lowest binding free energy were selected, and further visual assessment confirmed the interactions validity.

The proteins COX-1 and COX-2 were decided on due to their significant role in prostaglandin production, which is a key factor in inflammation. Inhibiting these enzymes reduces prostaglandin synthesis, aiding in pain and inflammation management, especially in conditions like arthritis. Furthermore, the MCF-7 (PDB code: 4XO6) and HepG2 (PDB code: 5EQG) receptors were selected for both in-silico and in-vitro studies due to their relevance in cancer research. MCF-7 is a breast cancer cell line expressing estrogen receptors, making it useful for breast cancer treatment studies, while the 4XO6 receptor is involved in crucial estrogen receptor signaling pathways in cancer progression. HepG2, a liver cancer cell line, was selected for its importance in liver cancer research, with the 5EQG receptor playing a key role in cancer development and drug response. These receptors were chosen to explore the therapeutic potential of the newly synthesized compounds through both docking and biological evaluations.

## Results and discussion

### Ligand description

The obtained Schiff base has the molecular formula C_25_H_19_Cl_2_N_3_O_2_ ((M.wt = 464.35 g/mol). The ligand was synthesized into a yellow powdery substance with a melting point recorded at 244 °C. The solubility of the HDN Schiff base was evaluated in some organic solvents. It dissolved well in DMSO and DMF, didn’t dissolve in EtOH or MeOH, and exhibited only slight solubility in acetone (Table 1).

^1^HNMR spectra of the HDN Schiff base were recorded utilizing the DMSO-d6 solvent. Table S1 and Figure S1 showcase the documented isomer shift values. The aromatic protons were identified in the range of δ = 6.32–9.23 ppm (m, 13H, ArH). The azomethine proton (-CH = N) was exhibited as a single peak at 10.90 ppm (s, 1H, CH = N). Phenolic OH was observed as a singlet peak at 12.40 ppm (s, 1H, OH). The singlet band seen at δ = 12.10 ppm is attributed to the protone of the N–NH group (s, 1H, N–NH). The band observed at δ = 11.60 ppm could be attributed to the 1H of the –NH-Ar group (s, 1H, -NH-Ar). The proton within the (O = CCH_2_-) group of the ligand exhibited at 3.78 ppm. The peaks at 2.50 ppm and 3.33 ppm were attributed to DMSO and HDO solvents, respectively.

The mass spectrum data aligns with the suggested formula of the hydrazide and the hydrazone. The molecular ion peak is noticed at m/z = 310.85 (2.69%) for diclofenac hydrazide and m/z = 464.65 (0.68%) for the synthesized ligand. These values correspond to the molecular weights of diclofenac hydrazide and the HDN hydrazone, respectively, as indicated in Figures S2 and S3. The expected fragmentation, which further confirm the structure, is listed in Table S2.

The FTIR spectra of diclofenac acid, diclofenac ethyl ester, diclofenac hydrazide, and the HDN ligand are detailed in Table 2 and visualized in Figures S4-S7. The intense broad bands noticed at 3328 cm^−1^ for diclofenac hydrazide and at 3457 cm^−1^ for the HDN ligand are associated with the vibration of the groups O–H, NH, and NH_2_. The stretching vibrational bands detected at 1638 cm^−1^ for diclofenac hydrazide and at 1649 cm^−1^ for the HDN ligand are likely credited to the stretching vibration of the C = O group—the azomethine group **υ**(C = N) of the formed Schiff base observed at 1591 cm^−1^.

Electronic spectra of the HDN Schiff base were obtained at ambient temperature utilizing a DMSO solution and are depicted in both Table 3 and Figure S14. The distinctive characteristic of the azomethine group (-CH = N-) and the absorption of the UV–visible spectrum is the presence of color, along with functional groups like C = O and C = C^36^. The identified bands attributed to the desired Schiff base can be linked to π → π* and n → π* transitions^37^. In the UV–vis spectra of the HDN Schiff base, four distinct bands are observed at wavelengths of (220 and 247 nm) and (317 and 344 nm), respectively. The noticed bands correspond to electronic transitions designated as π → π* and n → π*..

### Complexes obtained from the HDN Schiff base

The HDN were complexated with the target metal forming the corresponding complexes, which could be formulated as: [Co(DN)_2_], [Ni(DN)_2_, [Cu(DN)_2_]2.5H_2_O, [Gd(HDN)_2_(NO_3_)_2_]NO_3_.4H_2_O, [La(HDN)(NO_3_)_2_(H_2_O)_4_]NO_3_ and [Ag(DN)(H_2_O)] respectively. The elemental analysis results (C, H, N, M %) are consistent with the empirical formula as indicated in Table 1. All the synthesized compounds are consistent with distinctive colored powder. All the synthesized compounds gave a melting point at a range of 180- > 360 °C. The solubility of the metal complexes was evaluated in some organic solvents. It dissolved well in DMSO and DMF, didn’t dissolve in MeOH and EtOH, and exhibited only slight solubility in acetone. The molar conductivity of the complexes solutions was gauged using a 0.001 M DMSO solution. The complexes exhibited conductivities ranging from 12–26 Ω^−1^.cm^2^.mol^−1^, which denotes non-electrolytic behavior. On the other hand, the Gd(III) and La(III) complexes had conductivities of 78 and 72 Ω^−1^.cm^2^.mol^−1^, respectively, showing their electrolytic nature. For the Gd(III) and La(III) complexes, the charge balance is satisfied by the presence of nitrate ions (coordinated and as counter-ions). The observed molar conductance values further confirm their electrolytic nature, in line with the proposed formulations.

**Table 1:** Analytic facts of the formed compounds.

**Compounds**	**Mol. Wt**	**Color**	**Melting point** **(°C)**	**Elemental analysis** % Calc. % Foun. %				**Molar conductivity** **(ohm**^**−1**^**.cm**^**2**^**.mol**^**−1**^**)**
				C %	H %	N %	M %	
Ligand (HDN) C_25_H_19_Cl_2_N_3_O_2_	464.35	Yellow	244	64.67 64.59	4.12 4.21	9.05 9.23	–- –-	–-
[Co(DN)_2_]	985.61	Orange	320	60.93 60.66	3.68 3.44	8.53 8.24	5.98 6.05	15
[Ni(DN)_2_]	985.37	Yellowish Green	> 360	60.95 60.45	3.68 4.07	8.53 8.22	5.96 6.01	12
[Cu(DN)_2_]2.5H_2_O	1035.26	Dark green	296	58.01 58.33	3.99 4.36	8.12 8.44	6.14 6.24	16
[Gd(HDN)_2_(NO_3_)_2_]NO_3_.4H_2_O	1344.01	Light brown	189	44.68 44.35	3.45 3.67	9.38 9.11	11.70 12.36	78
[La(HDN)(NO_3_)_2_(H_2_O)_4_]NO_3_	861.32	Yellowish brown	180	34.86 35.22	3.16 3.44	9.76 9.95	16.13 16.34	72
[Ag(DN)(H_2_O)]	589.22	Dark yellow	280	50.96 50.45	3.42 3.17	7.13 6.75	18.31 –-	26

**Fig. 1:**
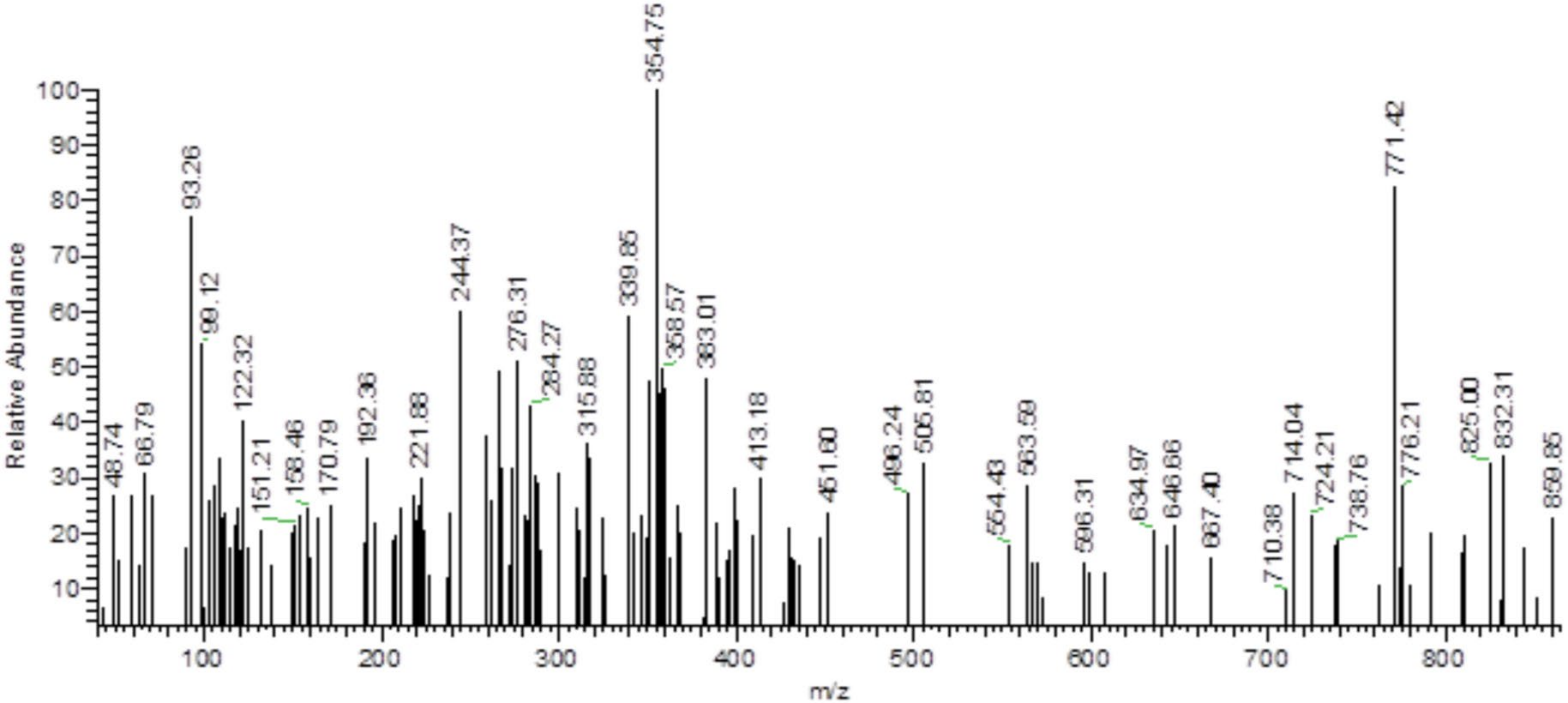
Mass spectrum of the La(III) complex.

The mass spectrum of the La(III) complex shows a molecular ion peak at **m/z = 859.85** with an intensity of 23.05%, aligning with the proposed molecular structure. This peak is in close agreement with the calculated molecular weight of the La complex, which supports the accuracy of the molecular formula (Fig. 1).

Additionally, the spectrum reveals details about the fragmentation pattern (illustrated in Fig. 1 in the document). The fragmentation proceeds in steps, reflecting the successive loss of particular groups from the complex, a pattern common among similar coordination complexes. This fragmentation sequence further supports the structural integrity of the La(III) complex, as the observed fragments match the expected breakdown of its coordination sphere and ligand components.

**Fig. 2:**
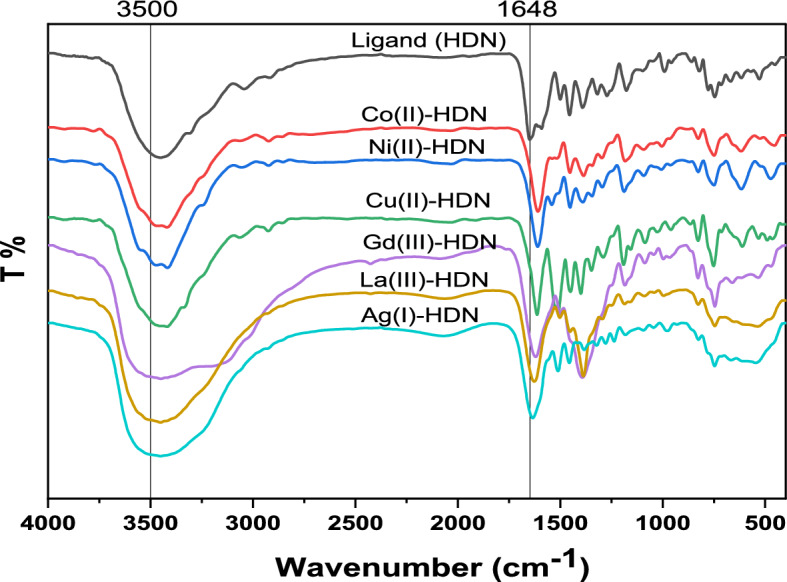
FTIR spectra of the established hydrazone and its metal complexes.

The complexes’ FTIR spectra are presented in Table 2 and illustrated in Fig. 2 and Figures S8-S13. The broad band observed at 3881–2477 cm^−1^ for the target compounds could be attributed to the stretching vibrations of the H_2_O, O–H, and N–H. The appearance of this band in all complexes implies that the prepared compounds contain coordinated and/or crystalline water. Each one of the prepared complexes exhibited new and relatively weak/medium/shoulder bands within the range of the M–O and M–N bands. More specifically, for the Co(II), Ni(II), Cu(II), Gd(III), La(III), and Ag(I) complexes, the M–O stretching band was noted at 615, 617, 612, 658, 652, and 665, respectively. In contrast, the M–N band was noticed at 525, 523, 532, 532, 536, and 546. The υ(C = O) appeared as a powerful band at 1649, 1605, 1611, 1613, 1619, 1626, and 1634 cm^−1^ for HDN ligand, Co(II), Ni(II), Co(II), Gd(III), La(III) and Ag(I) complexes, respectively. This characteristic underwent alteration upon complexation, signifying the involvement of this group in the chelation process. The υ(C = N) noticed at 1591 cm^−1^ for the HDN Schiff base exhibited a red shift in all synthesized complexes, indicating the involvement of these groups in the chelation process within the prepared complexes^38^. The downshift of ν(C = O) from 1649 cm⁻^1^ in the free ligand to ~ 1610–1626 cm⁻^1^ in the complexes confirms carbonyl coordination. Similarly, the azomethine ν(C = N) band at 1591 cm⁻^1^ exhibited a red shift upon complexation, indicating coordination through the azomethine nitrogen. The Gd(III) and La(III) complexes display distinct vibration frequencies that correspond to both ionic and coordinated nitrate groups. The bands observed around 1390 and 1387 cm^- 1^, respectively, imply the existence of ionic nitrate groups, attributable to the ʋ_3_​ vibration of the nitrate group with D_3h_ symmetry^39–41^. The Gd(III) and La(III) complexes display four bands at (1449, 1250 cm^−1^) and (1452, 1294 cm^−1^), respectively. These bands can be ascribed to the vibrational modes of coordinated nitrate groups with C_2v_ symmetry^42,43^ and sp^2^ hybridization. The magnitude of splitting of the higher energy bands (ʋ_4_—ʋ_1_) of the C_2v_ nitrate is approximately 199 and 158 cm^−1^, signaling that the nitrate groups are bonded to the metal atom in both bidentate and monodentate manners.

**Table 2:** FTIR spectral data of the formed ligand and its metal complexes (cm^−1^).

Compounds	υ(H_2_O), υ(OH) υ(NH)	ʋ(C = O)	ʋ(C = N)	Coordinated ʋ(NO_3_)			Ionic ʋ(NO_3_)	ʋ(M–O)	ʋ(M–N)
				ʋ4	ʋ1	∆ʋ	ʋ3		
Diclofenac acid	3442 m., 3328 m	1694 v.s	–-	–-	–-	–-	–-	–-	–-
Diclofenac ethyl ester	3299 s	1712 v.s	–-	–-	–-	–-	–-	–-	–-
Diclofenac hydrazide	3328 v.s	1638 v.s	–-	–-	–-	–-	–-	–-	–-
Ligand [HDN]	3457 v.s., br	1649 m	1591 w	–-	–-	–-	–-	–-	–-
[Co(DN)_2_]	3748–3092 br	1605 v.s	1539 w	–-	–-	–-	–-	615 m	525 w
[Ni(DN)_2_]	3744–3092 br	1611 v.s	1541 w	–-	–-	–-	–-	617 m	523 sh
[Cu(DN)_2_]2.5H_2_O	3753–3092 br	1613 v.s	1510 v.s	–-	–-	–-	–-	612 m	532 w
[Gd(HDN)_2_(NO_3_)_2_]NO_3_.4H_2_O	3881–2477 br	1619 v.s	1505 w	1449 sh	1250 w	199	1390 v.s	658 w	532 w
[La(HDN)(NO_3_)_2_(H_2_O)_4_]NO_3_	3782–2479 br	1626 v.s	1505 w	1452 w	1294 w	158	1387 v.s	652 sh	536 w
[Ag(DN)(H_2_O)]	3838–2534 br	1634 v.s	1511 m	–-	–-	–-	–-	665 w	546 w

v.s. = very strong, s. = strong, w. = weak, m. = medium, br. = broad, sh. = shoulder.

#### Magnetic moment and electronic spectra

The electronic spectra of these complexes were gauged in a solution with DMSO. The obtained data are displayed in Figures S14–20 and are compiled in Table 3. The electronic transitions observed for the synthesized Schiff base at 220 and 247 nm, as well as at 317 and 344 nm, corresponding to π → π* and n → π* transitions, experience a hypsochromic (blue) shift upon complexation. This shift is accompanied by either an increase (hyperchromic shift) or a decrease (hypochromic shift) in absorption intensity. This implies that the metal ion attaches to the Schiff base during the complexation process^44,45^.

**Table 3:** The electronic spectra and magnetic characteristics of the formed compounds.

Compounds	Peak		Postulated Electronic Transition	10 Dq		µ_eff_ _(B.M)_	Postulated Structure
	nm	cm^−1^		kJ/mol	(cm^−1^)		
Ligand (HDN)	220	45,454	π → π*	–-	–-	–-	–-
	247	40,486					
	317 344	31,546 29,070	n → π*				
[Co(DN)_2_]	218 247	45,872 40,486	π → π*	227	18,998	5.64	Octahedral
	350	28,571	n → π*				
	430	23,256	^4^T_1g_(F) → ^4^T_1g_(P)				
	658	15,198	^4^T_1g_(F)→^4^T_2g_(F)				
[Ni(DN)_2_]	221	45,249	π → π*	279	22,988	3.00	Octahedral
	247	40,486					
	345	28,985	n → π*				
	435	22,988	^3^A_2g_(F)→^3^T_2g_(F)				
[Cu(DN)_2_]2.5H_2_O	383	26,110	n → π*	181	15,151	2.22	distorted Octahedral
	589	16,978	^2^E_g_ → ^2^T_2g_				
	660	15,151	^2^E_g_ → ^2^A_1_				
[Gd(HDN)_2_(NO_3_)_2_]NO_3_.4H_2_O	408	24,510	n → π*	–-	–-	8.37	Square antiprismatic
	428	23,364					
[La(HDN)(NO_3_)_2_(H_2_O)_4_]NO_3_	274	36,496	π → π*	–-	–-	1.88	Tricapped trigonal prismatic
	310	32,258	n → π*				
	323	30,960					
	358	27,933					
	371	26,954					
	421	23,753	n → π*				
[Ag(DN)(H_2_O)]	414	24,155	n → π*	–-	–-	–-	Tetrahedral
	434	23,041					

The µ_eff_ value of the Co(II) complex is 5.64 BM. The magnetic moment value and electronic spectrum analysis confirmed the octahedral geometry around the Co(II) ion. The UV–Vis spectra of the cobalt (II) complex reveal significant peaks at (218, 247 nm) and 350 nm, indicating transitions from π → π* and n → π*, respectively. The octahedral geometry around the Co(II) ion was firmly verified by the transition bands that emerged at 430 and 658 nm, which could be designated as ^4^T_1g_(F) → ^4^ T_1g_(P) and ^4^T_1g_(F) → ^4^T_2g_(F).

The Ni(II) complex is paramagnetic, features an octahedral geometry, and exhibits a magnetic moment of 3.00 BM. Absorption bands are visible in the electronic spectra of this complex at (221, 247), 345, and 435 nm. These bands correspond to the transitions π → π*, n → π*, and ^3^**A**_**2g**_**(F)→ **^3^**T**_**2g**_**(F)**, respectively. These observations offer compelling evidence for the octahedral geometry around the Ni(II) ion. The [Cu(DN)_2_]2.5H_2_O complex, being paramagnetic, demonstrates an octahedral geometry and has a magnetic moment of 2.22 BM. Transitions from n→π*, ^**2**^**E**_**g**_**→**^**2**^**T**_**2g**_ and ^**2**^**E**_**g**_**→**^**2**^**A**_**1**_ are responsible for the distinct absorption bands observed at 383, 589, and 660 nm, respectively.

In the UV–Vis spectra, additional d–d and LMCT transitions further corroborate the proposed geometries, consistent with established assignments for octahedral Co(II)/Ni(II)/Cu(II) complexes.

The magnetic moments of the Gd(III) and La(III) complexes are 8.37 BM and 1.88 BM, respectively. While the La(III) complex exhibits absorption bands at 274 and (310, 323, 358, 371, and 421 nm) respectively, corresponding to π → π* and n → π* transition, the UV–Vis spectra of the Gd(III) complex display intense bands at 408 and 428 nm, ascribed to the n → π* transition. The tetrahedral geometry around Ag(I) ion is strongly supported by the absorption bands at 414 and 434 nm in the UV–Vis spectra of the Ag(I) complex, which are attributed to the n → π* transition.

#### Thermal analysis

**Fig. 3:**
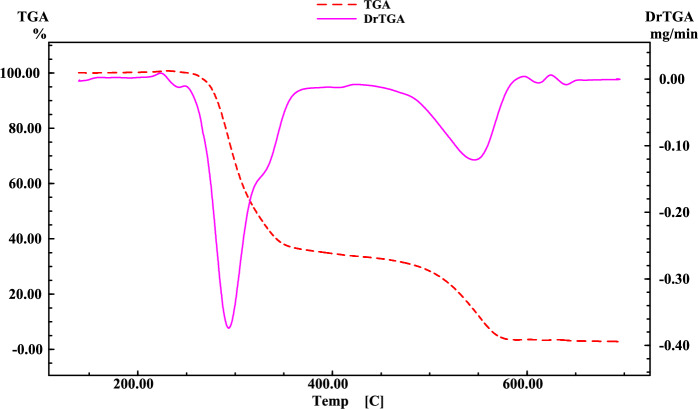
The thermal decomposition of the HDN ligand.

Thermogravimetric (TGA) and differential thermogravimetric (DTG) analyses were carried out on the HDN Schiff base and its metal complexes from room temperature up to 800 °C in a nitrogen environment. The various decomposition stages, temperature ranges, corresponding decomposition products, and both observed and theoretical weight losses of the metal complexes derived from the Schiff base are summarized in Table S3. The representative thermal curves of the HDN ligand and its metal complexes, depicted in Figs. 3 and **S21-S26**, provide insight into the thermal behavior.

The initial decomposition phase involves the loss of crystallization water. For [Cu(DN)_2_]·2.5H_2_O, dehydration occurred between 23–166 °C with a mass loss of 4.65% (calculated 4.35%) and a DTG peak at 42 °C. Similarly, [Gd(HDN)_2_(NO_3_)_2_]NO_3_·4H_2_O showed dehydration in the range of 32–115 °C with a mass loss of 5.13% (calculated 5.36%) and a DTG peak at 52 °C. The second stage involved the loss of coordinated water and nitrates, as seen in the La(III) and Ag(I) complexes. The La(III) complex lost water and nitrates between 131–436 °C with a mass loss of 30.58% (observed and calculated), with DTG peaks at 180 °C and 278 °C. In contrast, the Ag(I) complex exhibited mass losses of 16.48% (observed and calculated) between 29–416 °C, with DTG peaks at 275 °C and 363 °C.

The decomposition of the ligand began at varying temperatures for each complex. For [Co(DN)_2_], it started at 23 °C, for [Ni(DN)_2_] at 38 °C, for [Cu(DN)_2_] at 166 °C, for [Gd(HDN)_2_(NO_3_)_2_]NO_3_·4H_2_O at 115 °C, for [La(HDN)(NO_3_)_2_(H_2_O)_4_]NO_3_ at 436 °C, and for [Ag(DN)(H2O)] at 29 °C. The final decomposition phase started at 469 °C, 439 °C, 336 °C, 377 °C, 509 °C, and 416 °C for the Co(II), Ni(II), Cu(II), Gd(III), La(III), and Ag(I) complexes, respectively. The DTG peaks for the first ligand decomposition phase were observed at 317 °C, 327 °C, 293 °C, (202 °C and 277 °C), 509 °C, and (275 °C and 363 °C).

The corresponding mass losses were 28.03%, 25.25%, 19.59%, 30.71%, 52.68%, and 16.48%, closely matching the calculated values of 28.08%, 25.39%, 19.73%, 30.71%, 52.83%, and 16.48%. Nitrate group liberation occurred within the temperature ranges of 115–377 °C and 131–436 °C for the Gd(III) and La(III) complexes, respectively.

CoO, NiO, CuO, Gd, La, and Ag residues closely matched the calculated values, confirming the proposed decomposition patterns and formulas.

These results indicate that the La(III) complex had the highest overall thermal stability, while the Co(II) complex showed the lowest. Additionally, the Gd(III) complex exhibited the highest crystallization water stability, and the La(III) complex had the highest stability in coordinated water and nitrate loss.

#### Electron spin resonance (ESR) spectroscopy

**Fig. 4:**
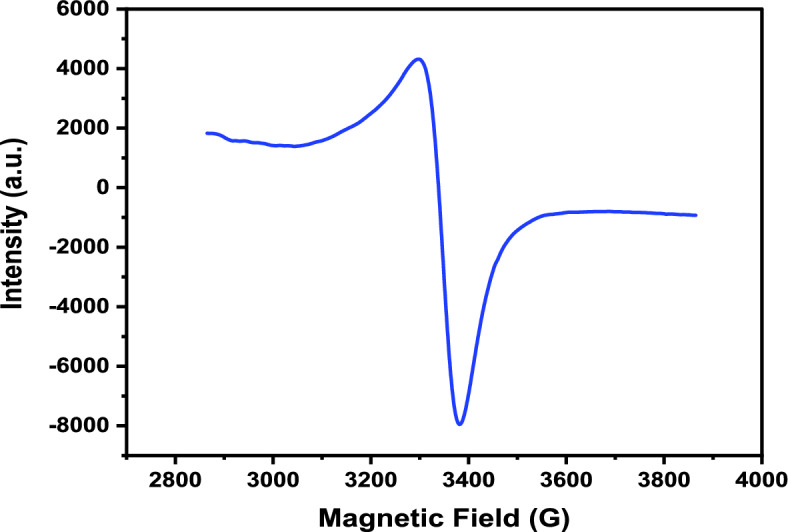
Electron spin resonance (ESR) of the Cu(II)-HDN complex.

The Electron Spin Resonance (ESR) spectra of the Cu(II)-HDN complex (Fig. 4 and Table S3) provide significant insight into the electronic environment and geometry around the Cu2⁺ ion. From the observed spectral data, it is possible to deduce that the complex adopts a distorted octahedral geometry, likely influenced by the Jahn–Teller effect, a common feature in copper(II) complexes^46^.

The anisotropic g-values, with g||= 2.06964 and g┴ = 2.10154, point to an axial distortion in the octahedral coordination environment. In a perfectly symmetrical octahedral field, the g-values would be nearly identical (isotropic). However, the disparity between g|| and g┴ suggests an elongation or compression along one axis, which is indicative of a Jahn–Teller distortion. In this case, the unpaired electron in the d⁹ configuration of Cu2⁺ likely resides in the d_x2−y2_​ orbital, causing a distortion where the axial bonds are longer than the equatorial bonds. This kind of distortion is common in Cu(II) complexes and aligns with the observed ESR data. The spin concentration of the complex, reported as 2.127 × 10^21^ spins/mole, is consistent with the presence of one unpaired electron per copper center. In a d⁹ system, such as Cu(II), the unpaired electron primarily resides in the d_x2−y2_​ orbital.

This configuration, combined with the Jahn–Teller distortion, stabilizes the complex and further supports the interpretation of a distorted octahedral geometry. The large hyperfine coupling constant (A = 965,275.60) reflects a strong interaction between the unpaired electron and the copper nucleus (I = 3/2 for Cu^2^⁺). This is characteristic of complexes where the electron density is concentrated in the d_x2−y2_​ orbital. The strength of this interaction suggests significant delocalization of the unpaired electron in the equatorial plane, with weaker interactions in the axial direction, further supporting the presence of axial elongation. The resonance magnetic field (H₀ = 3337.10) and the relatively narrow linewidth (ΔH = 81.70) indicate a well-ordered environment around the Cu^2^⁺ ions, with limited dipolar interactions between adjacent spins. This suggests that the unpaired electron is mainly localized in the equatorial plane of the distorted octahedral complex, reducing interactions along the axial direction. The effective magnetic field experienced by the Cu(II) centers (Hm = 8.00) aligns with this interpretation, further supporting the presence of a Jahn–Teller distorted octahedral geometry^47^.

The g-values and hyperfine coupling constant, when taken together, point to a Jahn–Teller distorted octahedral geometry. In Cu(II) complexes, the Jahn–Teller effect arises due to the d⁹ electronic configuration, which results in unequal electron distribution and thus geometric distortion to lower the system’s energy. Typically, this leads to elongation of the axial bonds, resulting in g||< g┴, as observed in the spectra. The Jahn–Teller distortion is well-documented in copper complexes and explains the anisotropic behavior seen in the ESR spectrum of the Cu(II)-HDN complex^48^.

The Jahn–Teller distortion identified in the ESR spectra of the Cu(II) complex may contribute to enhanced biological activity, as distortion can facilitate redox cycling and ROS generation^49^. Likewise, the nanocrystalline features observed in the XRD analysis (average crystallite size ~ 30 nm) may influence cellular uptake, thereby correlating structural features with biological potency^50^.

#### X-ray diffraction measurement

X-ray diffraction (XRD) is an essential method for analyzing the structural characteristics of materials. In the Cu(II)-HDN complex, the XRD analysis combined with theoretical modeling via EXPO2014 software provides deep insights into its crystal structure, particle size, and other key crystallographic parameters^51^.

**Fig. 5:**
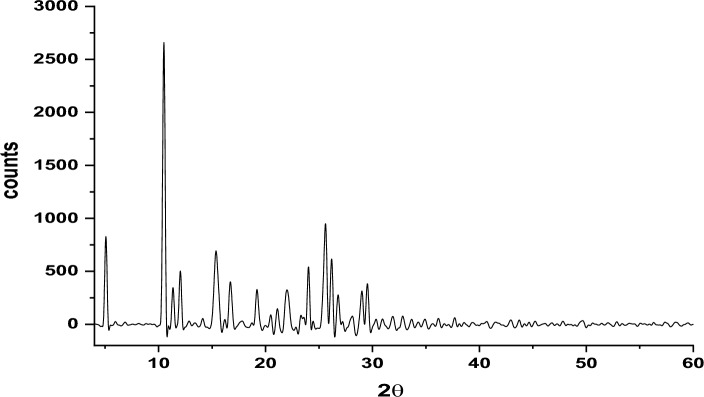
X-ray diffraction analysis of the Cu(II)-HDN complex.

The XRD data, illustrated in Fig. 5 and Table S5, show that the average particle size of the Cu(II)-HDN complex is 29.98 nm, confirming its classification as a nanomaterial. This nanoscale size is particularly important as nanomaterials are known for their enhanced properties, such as increased surface area and higher reactivity, making them suitable for various applications, including catalysis and materials science.

Key indicators of the crystalline quality, such as the Full Width at Half Maximum (FWHM) values of the diffraction peaks, the associated 2θ angles, and crystallite sizes, provide information on the particle size distribution and structural integrity. The observed strain (δ) values, ranging from 4.25 × 10⁻^4^ to 5.41 × 10⁻^3^, suggest slight internal distortions within the crystal lattice, a feature commonly seen in nanoscale materials.

**Fig. 6:**
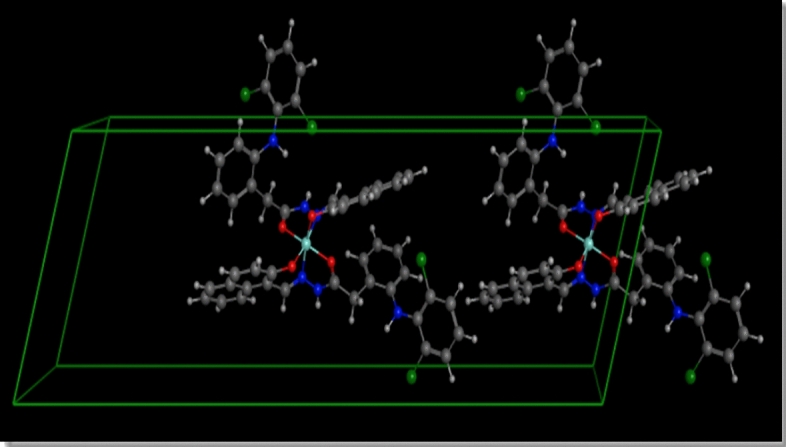
The unit cell of the Cu(II)-HDN complex.

**Fig. 7:**
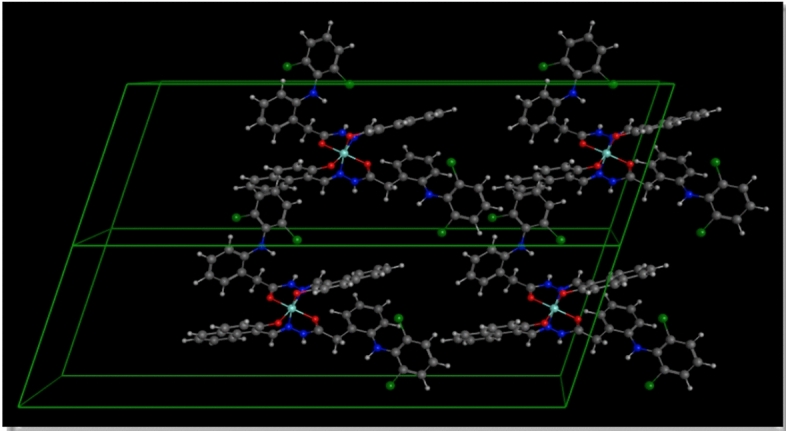
The package 1 2 1 crystal lattice for Cu(II)-HDN complex.

**Fig. 8:**
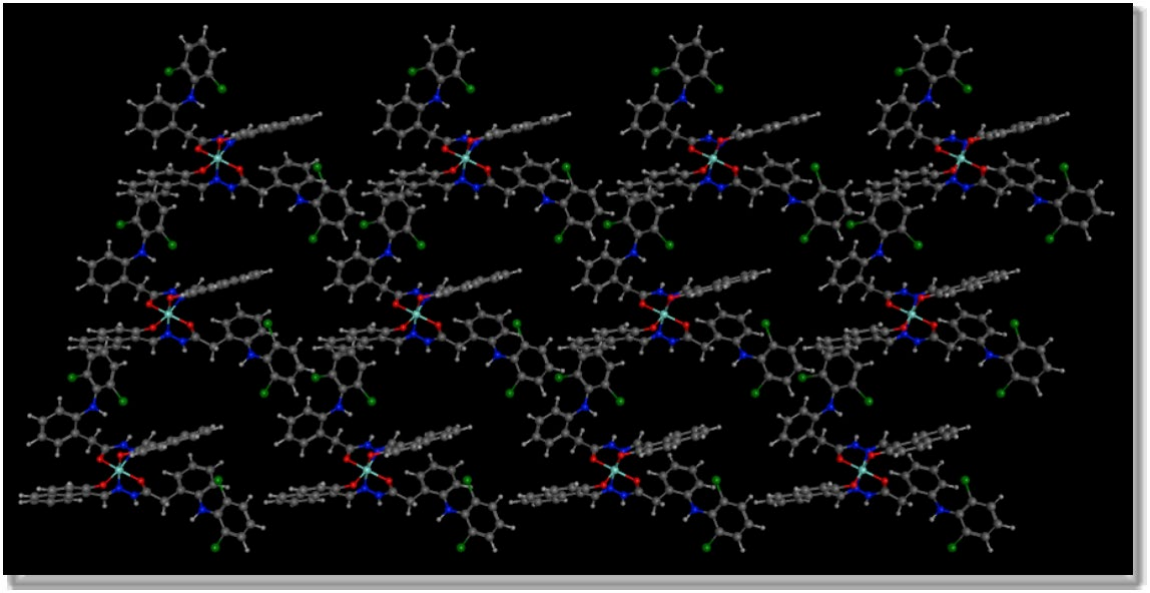
The 2D package of the crystal lattice for Cu(II)-HDN complex.

The theoretical modeling with EXPO2014, summarized in Table S6, reveals that the Cu(II)-HDN complex crystallizes in a triclinic crystal system with the P1 space group. This flexible lattice configuration allows for variable unit cell dimensions, which are: a = 18.22 Å, b = 11.99 Å, c = 8.59 Å, α = 99.09°, β = 95.89°, and γ = 72.41°. These parameters describe the three-dimensional atomic arrangement within the crystal. The calculated unit cell volume is 1763.18 Å^3^, with a per-atom volume of 27.46 Å^3^, and a Z-value of 2, indicating two formula units per unit cell. The use of EXPO2014 also provides visual representations of the unit cell structure and crystal packing, as seen in Figs. 6, 7 and 8. These visualizations clarify how the molecules are arranged and how the copper ion interacts with the HDN ligands within the solid-state structure^25^.

In summary, the combination of XRD data and EXPO2014 modeling highlights several critical aspects of the Cu(II)-HDN complex. The nanometer-scale particle size suggests unique physical and chemical properties, which could enhance the complex’s performance in applications where nanomaterials are advantageous. Despite its small size, the minimal internal strain suggests a well-ordered crystalline structure, contributing to its stability and potential reactivity. The crystallographic data, particularly the triclinic structure and P1 space group, provide a detailed understanding of the complex’s molecular organization and intermolecular interactions, which are essential for predicting its behavior in various environments.

**Fig. 9:**
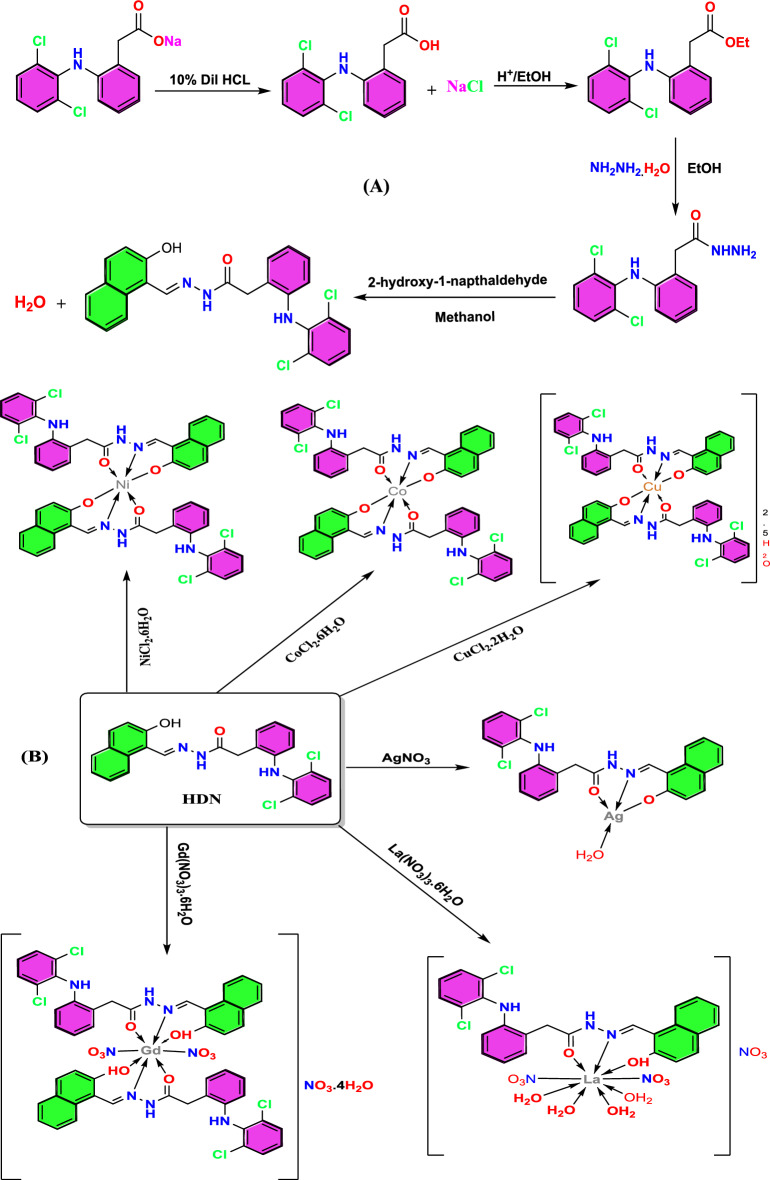
The postulated structure of the HDN ligand (**A**) and its complexes (**B**).

The synthesis pathway for the HDN ligand is outlined in **Scheme 1.** The structure of the prepared complexes could be inferred from the results of the physico-chemical analysis, which encompassed elemental analysis, conductivity, FTIR, electronic spectra, and thermal analysis, as depicted in Fig. 9.

### *Assessment of the anti-inflammatory activity of some selected compounds *in vitro* utilizing enzyme-linked immunosorbent assay (ELISA) methods*

**Fig. 10:**
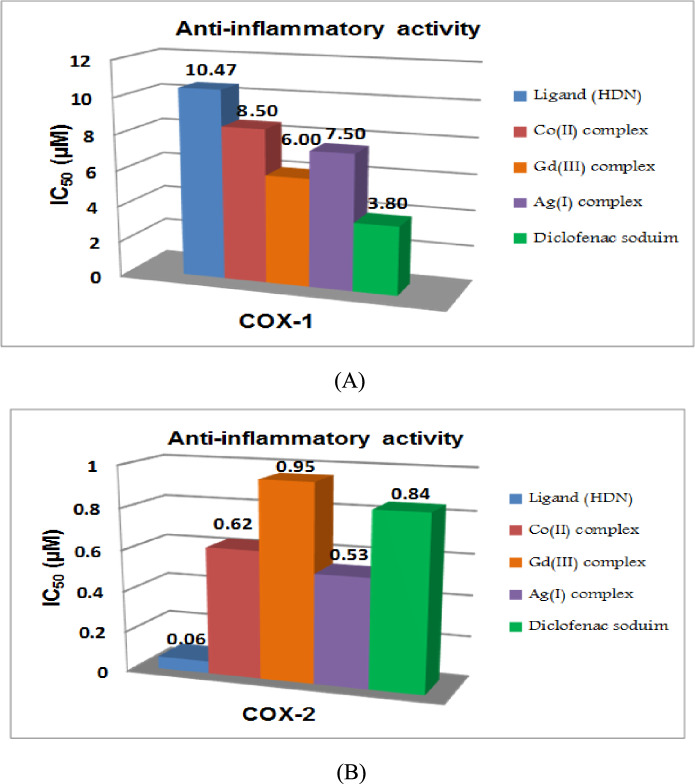
Inhibition of COX-1 (**A**) and COX-2 in vitro (**B**).

The anti-inflammatory potential of selected compounds (Table S7 and Fig. 10), including the HDN ligand and its metal complexes with Co(II), Gd(III), and Ag(I), was assessed through their inhibition of cyclooxygenase enzymes COX-1 and COX-2 using enzyme-linked immunosorbent assay (ELISA) methods^52^. These enzymes are crucial targets in the development of anti-inflammatory agents due to their roles in prostaglandin synthesis, with COX-1 associated with normal physiological functions and COX-2 predominantly involved in inflammation.

The IC_50_ values, which represent the concentration required to inhibit 50% of enzyme activity, revealed that the selected compounds exhibit varying levels of inhibition against COX-1 and COX-2. The IC_50_ values for COX-1 inhibition ranged from 6.00 µM (Gd(III)-HDN complex) to 10.47 µM (HDN ligand). For COX-2, the IC_50_ values were notably lower, ranging from 0.06 µM (HDN ligand) to 0.95 µM (Gd(III)-HDN complex), indicating stronger inhibition of the COX-2 enzyme across all compounds tested.

The COX-2 selectivity index (S.I.)**,** which is the ratio of COX-1 IC_50_ to COX-2 IC_50_, is a crucial parameter for determining how selectively a compound inhibits COX-2 over COX-1. This selectivity is desirable because COX-2 inhibition is linked to anti-inflammatory effects, whereas COX-1 inhibition can lead to gastrointestinal side effects. The HDN ligand showed the highest selectivity for COX-2 with an S.I. of 174.50, which was significantly higher than those of its metal complexes: Co(II) (13.71), Gd(III) (6.31), and Ag(I) (14.15).

The tested compounds were compared with standard anti-inflammatory drugs, including diclofenac sodium, rofecoxib, indomethacin, and celecoxib. Among these, rofecoxib exhibited the highest selectivity for COX-2 (S.I. = 725.00), followed by celecoxib (S.I. = 290.00). However, the HDN ligand showed better selectivity compared to diclofenac sodium (S.I. = 4.52) and indomethacin (S.I. = 1.25). This indicates that the HDN ligand is a promising candidate for selective COX-2 inhibition, potentially reducing inflammation while minimizing side effects associated with COX-1 inhibition.

The data suggest that the HDN ligand is the most potent COX-2 inhibitor among the compounds tested, with an IC_50_ of 0.06 µM and high selectivity. While less selective than the ligand, the metal complexes still demonstrated stronger COX-2 inhibition compared to diclofenac sodium, a commonly used nonsteroidal anti-inflammatory drug (NSAID). Except for the Gd(III) complex, all derivatives exhibited higher potency as COX-2 inhibitors than diclofenac sodium.

In conclusion, the study highlights the strong anti-inflammatory potential of the HDN ligand, particularly its high COX-2 selectivity, making it a promising candidate for further development as a selective COX-2 inhibitor. Its performance in comparison to standard drugs like diclofenac sodium and indomethacin supports the potential therapeutic value of these compounds, especially in conditions requiring targeted anti-inflammatory activity with minimal side effects.

It should be noted that COX-2 inhibitors, despite their selectivity, can carry potential cardiovascular liabilities. Although our findings highlight selective inhibition in vitro, future in vivo studies are required to assess off-target effects, particularly cardiovascular safety. These aspects are crucial before any clinical translation^53^.

### *Evaluation of *in vitro* antitumor activity of some selected compounds utilizing the MTT assay protocol:*

The antitumor effects of the newly synthesized HDN ligand and its metal complexes with Cu(II) and Gd(III) were tested against MCF-7 (breast cancer) and HepG-2 (liver cancer) cell lines using the MTT assay^28–30^.. This method measures cell viability based on mitochondrial activity, clearly indicating each compound’s cytotoxic potential. The key findings from these tests are outlined in Tables 4**, **S8, and S9 and illustrated in Figures S21-S31.

**Table 4:** The in vitro IC_50_ values of the HDN Schiff base, Cu(II)-HDN and Gd(III)-HDN complexes against MCF-7 and HepG-2 cell lines.

**Compounds**	IC_50_ (µM)		References
	**HepG-2**	**MCF-7**	
Ligand [HDN]	**151.80**	**177.90**	**This work**
Cu(II)-HDN	**1.00**	**0.65**	
Gd(III)-HDN	**2.47**	**0.80**	
Diclofenac sodium	**147.33**	**1788.79**	^54,64^
Ibuprofen	**–-**	**5061.17**	^54^
Diclofenac Zn(II)-nicotinamide	**–-**	**298.05**	
Ibuprofen Zn(II)-nicotinamide	**–-**	**1770.56**	
5- Fluorouracil	**32.53**	**3.95**	^55,56^
Cisplatin	**8.45**	**15.24**	^57,58^

Among the tested compounds, the Cu(II) complex exhibited the highest cytotoxic activity, with IC_50_ values of 0.65 µM against MCF-7 and 1.00 µM against HepG-2, demonstrating its strong inhibitory effects even at low concentrations. The Gd(III) complex also showed significant potency, with IC_50_ values of 0.80 µM for MCF-7 and 2.47 µM for HepG-2. In contrast, the HDN ligand alone was considerably less effective, with IC_50_ values of 177.90 µM (MCF-7) and 151.80 µM (HepG-2), suggesting that metal coordination greatly enhances its antitumor efficacy.

The concentration-dependent inhibition, as presented in Tables S8 and S9, further illustrates the effectiveness of the Cu(II) and Gd(III) complexes. At a concentration of 10 µM, both complexes achieved nearly complete inhibition of MCF-7 and HepG-2 cells, with inhibition rates exceeding 95%. Even at lower concentrations, the Cu(II) complex maintained substantial activity, inhibiting 63.27% of MCF-7 cells and 35.16% of HepG-2 cells at 0.62 µM. In contrast, the HDN ligand displayed much lower inhibition rates, dropping to 7.98% for MCF-7 and 12.93% for HepG-2 at similar concentrations. These results clearly demonstrate the enhanced cytotoxicity brought about by metal coordination.

When compared with anti-inflammatory drugs like diclofenac sodium and ibuprofen, along with their zinc(II) derivatives (Table 4). Diclofenac sodium exhibited IC50 values of 1788.79 µM for MCF-7 and 147.33 µM for HepG-2, which were significantly higher than those of the Cu(II) and Gd(III) complexes. Even the zinc(II)-nicotinamide complexes of diclofenac and ibuprofen, known for their improved anticancer properties, were much less effective than the synthesized metal complexes, with IC50 values as follows: Diclofenac Zn(II)-nicotinamide: 298.05 µM (MCF-7). Ibuprofen Zn(II)-nicotinamide: 1770.56 µM (MCF-7). This comparison emphasizes the much greater potency of the Cu(II)-HDN and Gd(III)-HDN complexes against cancer cells, especially MCF-7^54^.

The performance of the synthesized compounds was also compared to standard anticancer drugs, such as 5-fluorouracil^55,56^ and cisplatin^57,58^, the Cu(II) and Gd(III) complexes showed superior results, particularly against breast cancer cells (Table 4). For instance, 5-fluorouracil had an IC_50_ of 3.95 µM against MCF-7 and 32.53 µM against HepG-2. Cisplatin showed an IC_50_ of 15.24 µM for MCF-7 and 8.45 µM for HepG-2. This indicates that both metal complexes, particularly the Cu(II) complex, are far more effective than 5-fluorouracil and cisplatin in inhibiting cancer cell growth, especially in breast cancer cells, where the Cu(II) complex is approximately six times more potent than 5-fluorouracil.

Compared with other reported NSAID-based metal complexes, the present Cu(II)– and Gd(III)–HDN complexes display markedly enhanced potency against MCF-7 and HepG-2 cells. For example, Zn(II)–diclofenac derivatives typically exhibit IC_50_ values in the range of 200–300 µM, whereas our Cu(II) complex was active at submicromolar concentrations. This highlights the novelty of the present scaffold, especially in the context of Schiff base hydrazones derived from diclofenac^13,59^.

The results of the MTT assay show that the Cu(II) and Gd(III) complexes exhibit significant antitumor activity, with particularly strong effects on MCF-7 and HepG-2 cell lines. These complexes outperform the HDN ligand alone and show greater potency than widely used chemotherapeutic agents like 5-fluorouracil and cisplatin. Additionally, they are far more effective than anti-inflammatory drug derivatives such as diclofenac and ibuprofen complexes. The exceptional activity of the Cu(II)-HDN complex, in particular, makes it a promising candidate for further research and development as a potential cancer therapy. Future studies should explore its mode of action and evaluate its efficacy in in vivo models.

The markedly higher cytotoxicity of the Cu(II) and Gd(III) complexes may be attributed to their electronic structures and geometries. The Jahn–Teller distorted octahedral Cu(II) center facilitates redox cycling, whereas the larger ionic radius of Gd(III) enables favorable ligand coordination and cellular interaction. These correlations suggest that geometric/electronic parameters play a key role in modulating biological activity^60^.

We acknowledge that Schiff bases may undergo hydrolytic degradation under biological conditions^61^. While direct stability assays were not performed in this study, thermal analysis, molar conductance, and solubility data suggest reasonable stability of the complexes. Nonetheless, dedicated stability investigations in biological media will be essential in future work.

While the current study did not experimentally determine the mechanism of cytotoxicity, several plausible pathways can be proposed based on literature. Cu(II) and other transition-metal complexes are often associated with reactive oxygen species (ROS) generation, mitochondrial dysfunction, and DNA/protein binding, all of which can trigger apoptosis. Furthermore, COX-2 inhibition may contribute indirectly to anti-cancer effects through modulation of prostaglandin-mediated signaling. These mechanistic hypotheses will require validation in future dedicated apoptosis and ROS assays^49,62,63^.

### *Predicting bioactivity, pharmacokinetic characteristics, and physicochemical descriptors *in silico* for the prepared compounds*

Using the SwissADME web tool, the synthesized compounds’ physicochemical properties and pharmacokinetic characteristics—including the HDN Schiff base, its metal complexes, and the standard drug diclofenac sodium—were predicted^31^. This analysis focused on essential parameters such as topological polar surface area (TPSA), lipophilicity (LogP), and water solubility (LogS), in addition to pharmacokinetic attributes like gastrointestinal (GI) absorption and blood–brain barrier (BBB) permeability (Table 5).

**Table 5:** Predicted physicochemical descriptors of the target compounds.

**Compound**	**Mol. Wt**	**Heavy atoms**	**Rotatable bonds**	**H-bond donors**	**H-bond acceptors**	**Fraction Csp3**	**Silicos-IT (water)**	**WLOGP**	**XLogP3**	**Molar refractivity**	**Topological polar surface area (TPSA)**	**GI absorption**	**BBB** p**ermeant**	**P-gp substrate**
Diclofenac sodium	318.13	20	5	1	2	0.07	−6.35	4.29	4.49	76.01	38.33	High	Yes	No
ligand [HDN]	464.35	32	7	3	3	0.04	−10.17	6.29	6.48	131.29	73.72	High	No	Yes
[Co(DN)_2_]	985.61	65	18	4	6	0.04	−20.66	12.85	13.54	259.79	125.44	Low	No	Yes
[Ni(DN)_2_]	985.37	65	18	4	6	0.04	−20.66	12.85	13.54	259.79	125.44	Low	No	Yes
[Cu(DN)_2_]2.5H_2_O	1035.26	65	18	4	6	0.04	−20.67	12.85	13.54	259.79	125.44	Low	No	Yes
[Gd(HDN)_2_(NO_3_)_2_]NO_3_.4H_2_O	1344.01	67	14	8	8	0.04	−10.17	12.45	11.97	268.67	165.90	Low	No	Yes
[La(HDN)(NO_3_)_2_(H_2_O)_4_]NO_3_	861.32	48	10	12	18	0.04	−10.17	3.94	1.70	166.12	295.54	Low	No	Yes
[Ag(DN)(H_2_O)]	589.22	36	7	6	6	0.04	−10.17	6.10	5.04	140.43	101.41	Low	No	No

**Fig. 11:**
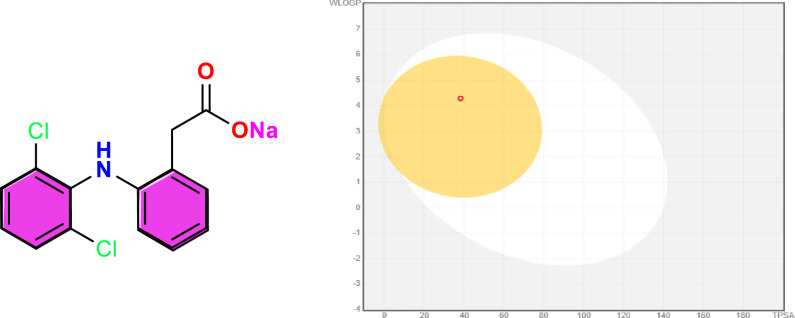
BOILED-Egg model for the diclofenac sodium. GI absorption (White colour)**: High**; BBB permeant (Yellow colour): **Yes.**

**Fig. 12:**
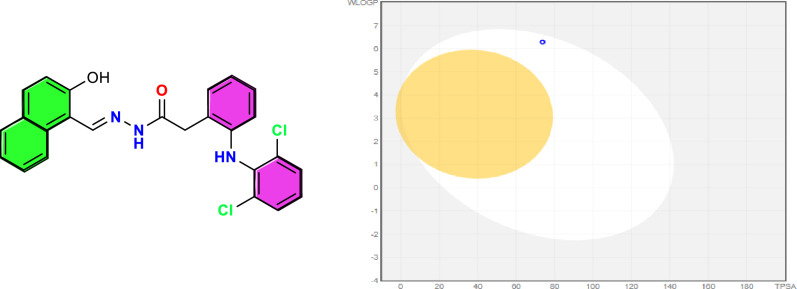
BOILED-Egg model for the HDN ligand. GI absorption (White colour)**: High**; BBB permeant (Yellow colour): **No.**

The BOILED-Egg model was applied to evaluate the likelihood of these compounds penetrating the BBB and their passive GI absorption. In this model^65,66^. The yellow region signifies a high probability of brain penetration. The white region indicates potential for passive GI absorption. The HDN ligand was predicted to have high GI absorption, while its metal complexes exhibited lower absorption rates (Figs. 11 and 12). Notably, none of the complexes were found to cross the BBB, which may reduce central nervous system-related side effects.

Veber’s rule assessed the compounds’ drug-likeness, suggesting that compounds with TPSA ≤ 140 Å^2^ and fewer than ten rotatable bonds exhibit good bioavailability^67,68^. The HDN ligand showed no violations of these criteria, supporting its potential as a candidate for further bioactivity studies.

In terms of lipophilicity, the n-octanol/water partition coefficient (LogP) values of the HDN ligand and its metal complexes were found to be higher than that of diclofenac sodium (4.29), except for the La(III) complex. The LogP values of the HDN Schiff base and its Cu(II), Co(II), Ni(II), Gd(III), and Ag(I) complexes ranged from 6.10 to 12.85, indicating that these compounds are more lipophilic than diclofenac sodium, which may influence their permeability and absorption properties.

The HDN ligand and its complexes display promising pharmacokinetic characteristics, with high GI absorption and favorable physicochemical profiles. The enhanced lipophilicity and other key descriptors, such as TPSA and molar refractivity, suggest that these compounds are well-suited for further investigation in bioactivity studies.

### Docking and simulation work

The molecular docking studies conducted on the HDN Schiff base and its metal complexes, including Ag(I)-HDN and Cu(II)-HDN, revealed important interactions with several key proteins, such as COX-2 (PDB code: 5ikt), HepG-2 (PDB code: 5eqg), and MCF-7 (PDB code: 4xo6). These analyses provided valuable insights into the prepared compounds’ binding modes, interaction energies, and potential bioactivity.

#### Interaction with COX-2

**Fig. 13:**
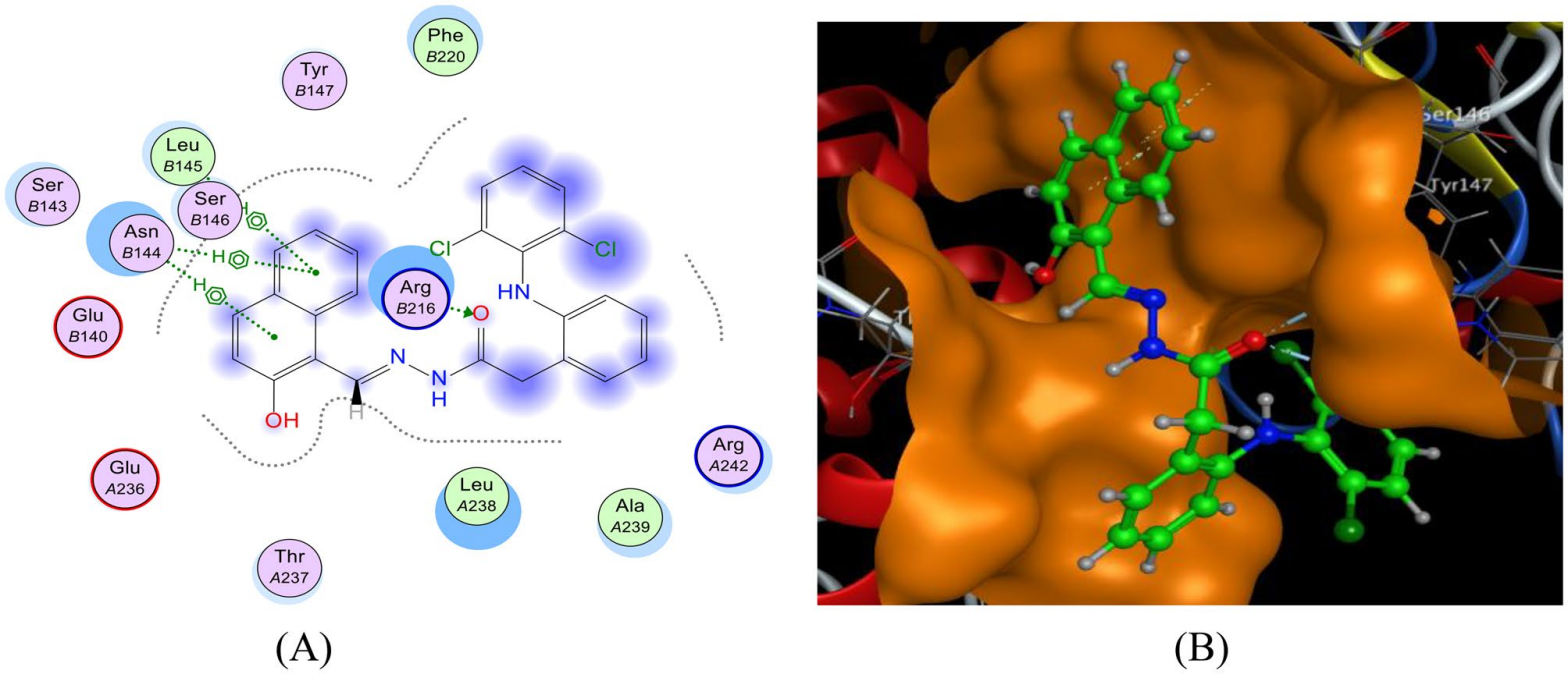
The docking of the HDN ligand with COX-2: 5ikt receptor: (**A**) 2D and (**B**) 3D view.

**Fig. 14:**
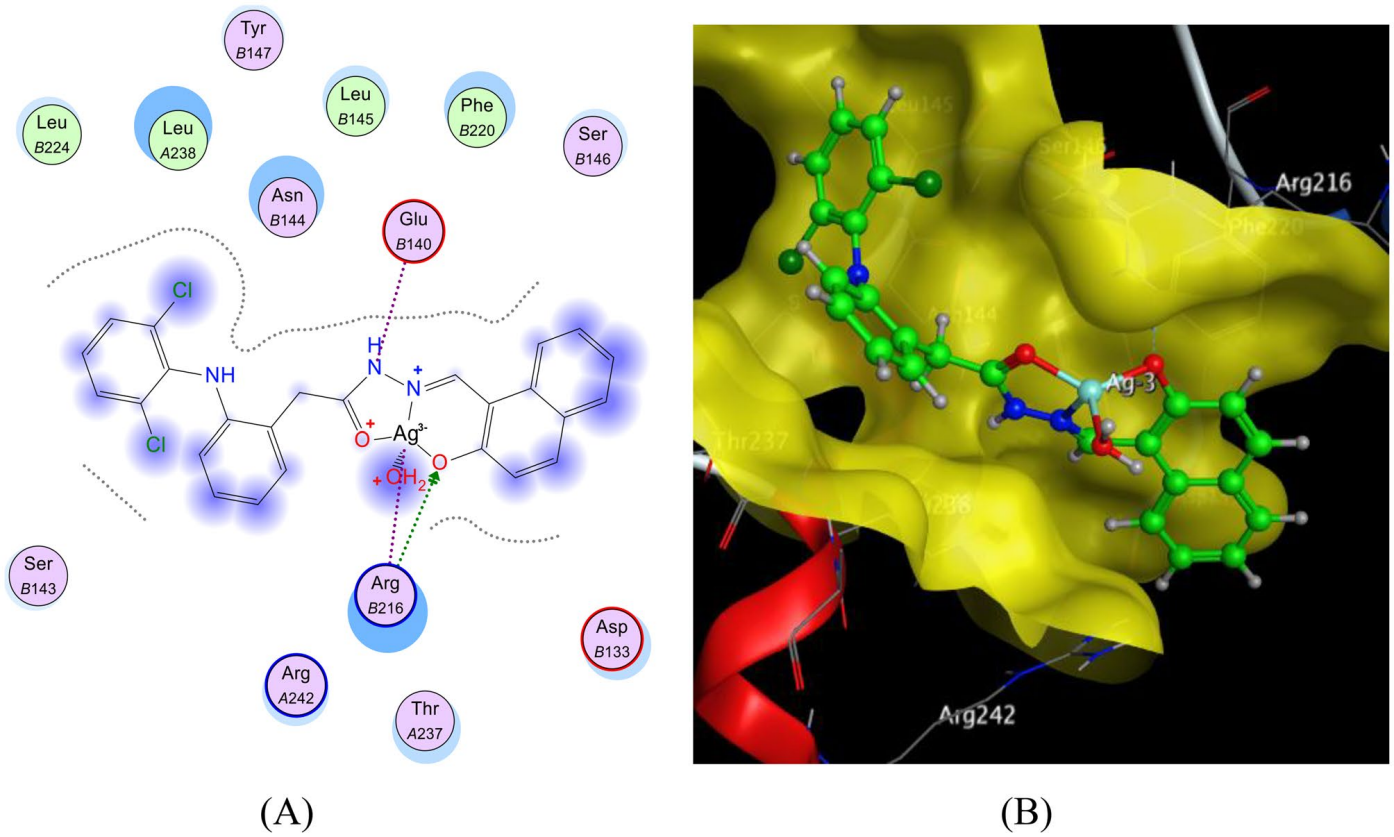
The docking of the Ag(I)-HDN complex with COX-2: 5ikt receptor: (**A**) 2D and (**B**) 3D view.

The docking of the HDN ligand and Ag(I)-HDN complex with COX-2 (Figs. 13 and 14) demonstrated the presence of hydrogen and ionic bonds. The HDN ligand formed hydrogen bonds with ARG216(B), while the Ag(I)-HDN complex exhibited both hydrogen and ionic interactions, including bonds with ARG216(B) and GLU140(B). The HDN and Ag(I)-HDN binding energies were −22.87 kcal/mol and −22.60 kcal/mol, respectively, indicating strong interactions and a high likelihood of inhibitory effects against COX-2 (Table 6). This aligns with the in vitro results, where the HDN ligand showed greater activity than the Ag(I) complex.

**Table 6:** The observed interaction parameters of the HDN ligand and Ag(I)-HDN complex with COX-2 [PDB code: 5ikt].

Ligand	Ligand site	Receptor site	Interaction type	Distance (Å)	Energy (kcal/mol)	Rmsd	Binding Energy (kcal/mol)
HDN	O8	NH1 ARG216(B)	H-A	2.89	−3.4	1.74	−22.87
	O8	NH2 ARG216(B)	H-A	3.02	−3.2		
	6-ring	Cα ASN144(B)	Pi-H	4.42	−0.7		
	6-ring	Cα ASN144(B)	Pi-H	4.63	−0.8		
	6-ring	N LUE145(B)	Pi-H	4.46	−0.9		
Ag(I)-HDN	O17	NH1 ARG216(B)	H-A	2.74	−1.3	1.88	−22.60
	N19	Oε2 GLU140(B)	Ionic	3.83	−0.9		
	Ag51	NH1 ARG216(B)	Ionic	3.16	−3.5		

#### Interaction with HepG-2

**Fig. 15:**
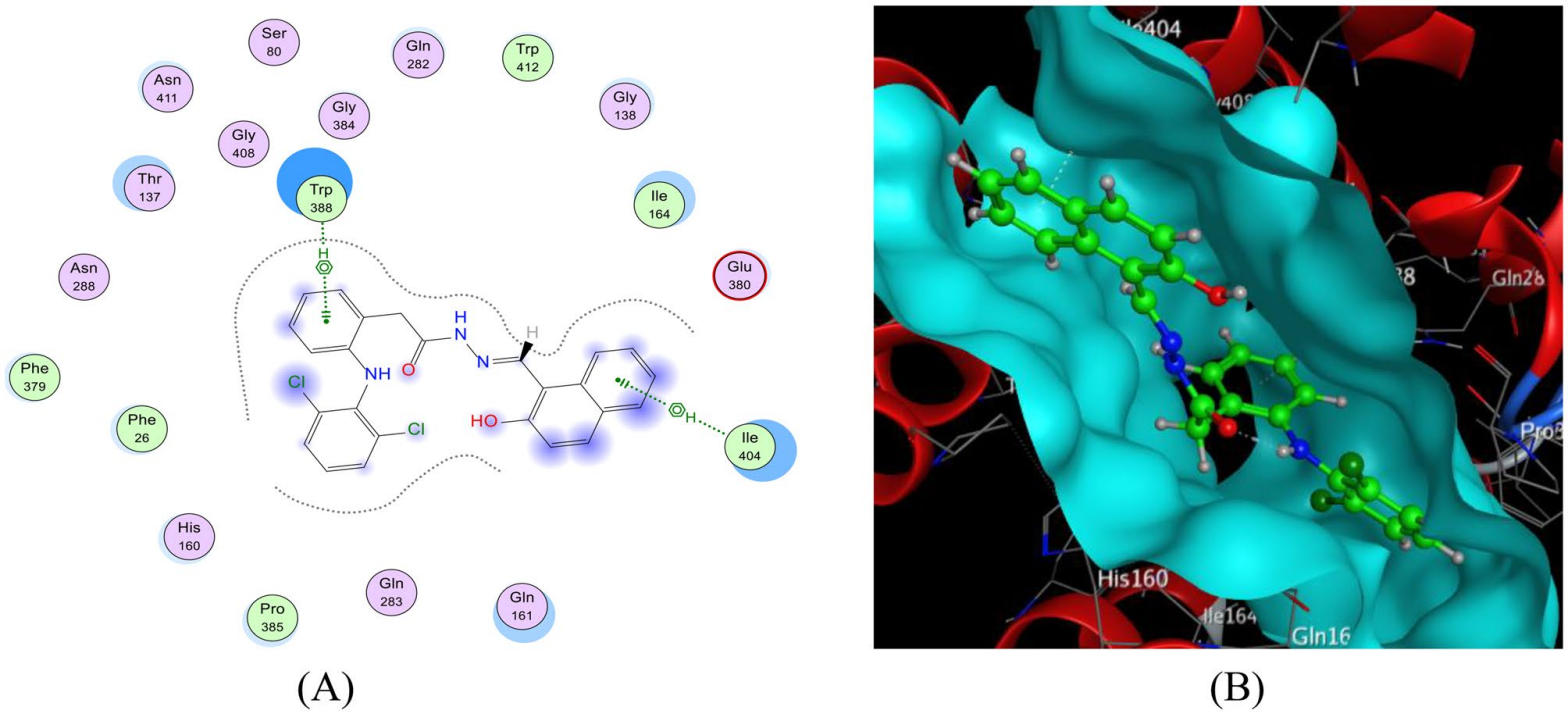
The docking of the HDN ligand with HepG-2: 5eqg receptor: (**A**) 2D and (**B**) 3D view.

**Fig. 16:**
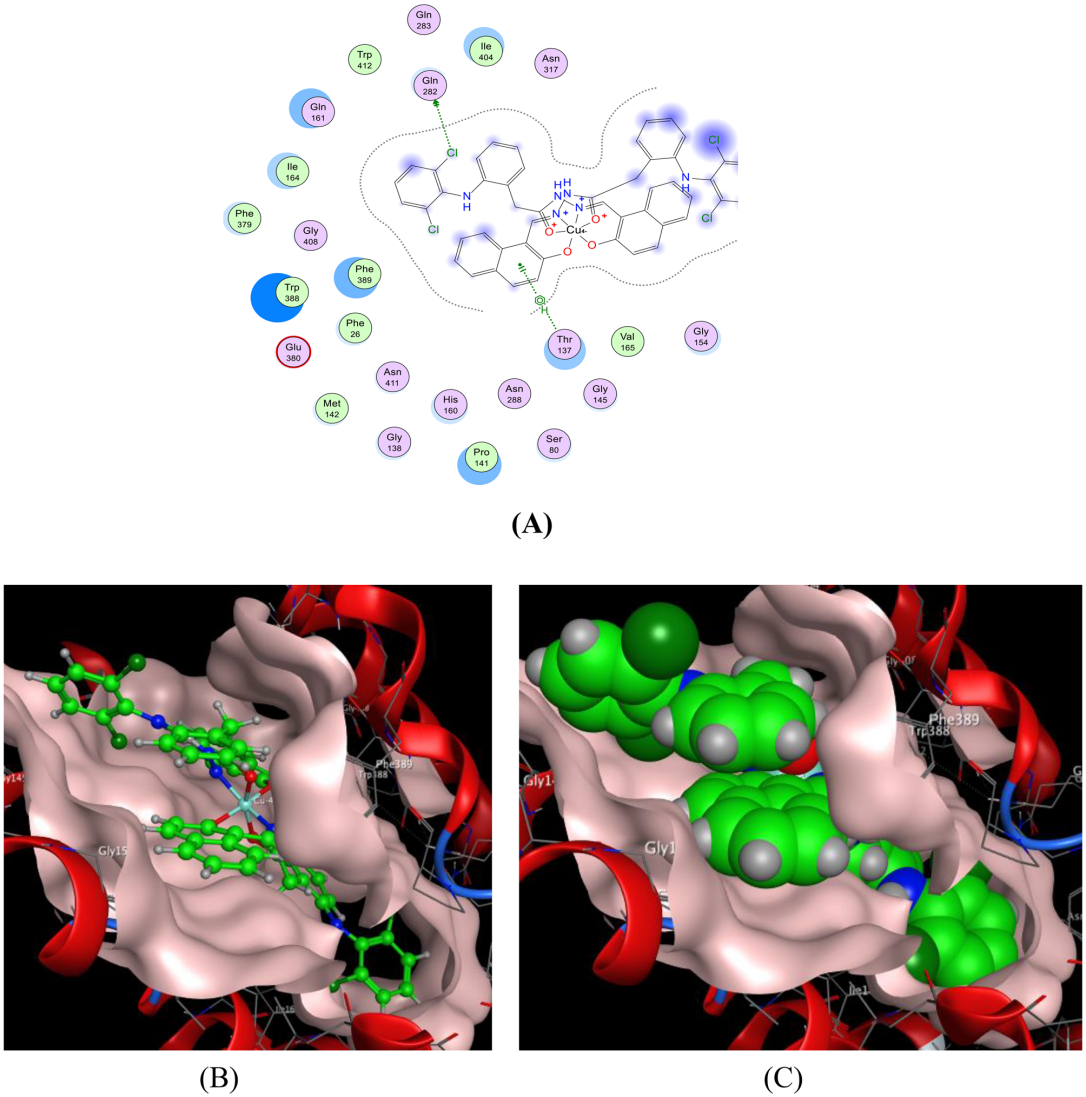
The docking of the Cu(II)-HDN complex with HepG-2: 5eqg receptor: (**A**) 2D, (**B**) 3D view and (**C**) 3D view.

Docking results with the HepG-2 receptor (Figs. 15 and 16) highlighted significant binding interactions for both the HDN ligand and the Cu(II)-HDN complex. The HDN ligand formed hydrogen bonds with TRP388(A) and ILE404(A), whereas the Cu(II) complex interacted with GLN282(A) and THR137(A), demonstrating a mixture of hydrogen and Pi-H interactions. The binding energies were −32.56 kcal/mol for HDN and −31.76 kcal/mol for Cu(II)-HDN (Table 7). Interestingly, despite the similar docking energies, the in vitro results showed that the Cu(II) complex had superior cytotoxic activity compared to the HDN ligand, suggesting that Cu(II)-HDN may have enhanced biological activity in live systems.

**Table 7:** The evident interaction parameters for some receptors with the target compounds.

Receptor	Ligand	Ligand site	Receptor site	Interaction type	Distance (Å)	Energy (kcal/mol)	Rmsd	Binding energy (kcal/mol)
**HepG-2** (PDB code 5eqg)	HDN	6-ring	Nε1 TRP388(A)	Pi-H	4.15	−0.8	1.31	−32.56
		6-ring	Cγ2 ILE404(A)	Pi-H	3.85	−0.8		
	Cu(II)-HDN	C124	Oε1 GLN282(A)	H–D	3.03	−0.1	1.56	−31.76
		6-ring	Oγ1 THR137(A)	Pi-H	4.02	−1.2		

#### Interaction with MCF-7

**Fig. 17:**
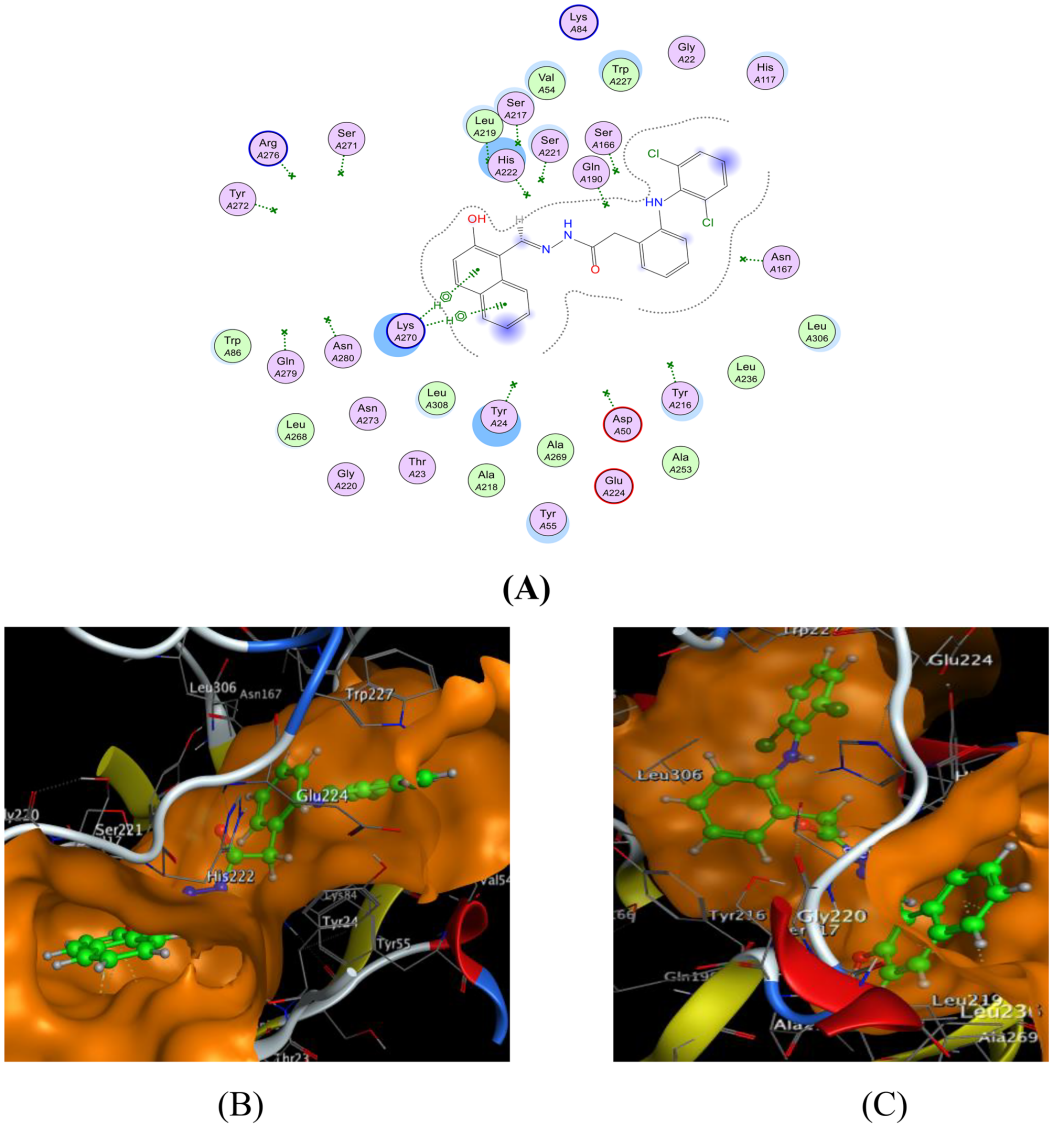
The docking of the HDN ligand with MCF-7:4xo6 receptor: (**A**) 2D, (**B**) 3D view and (**C**) 3D view.

**Fig. 18:**
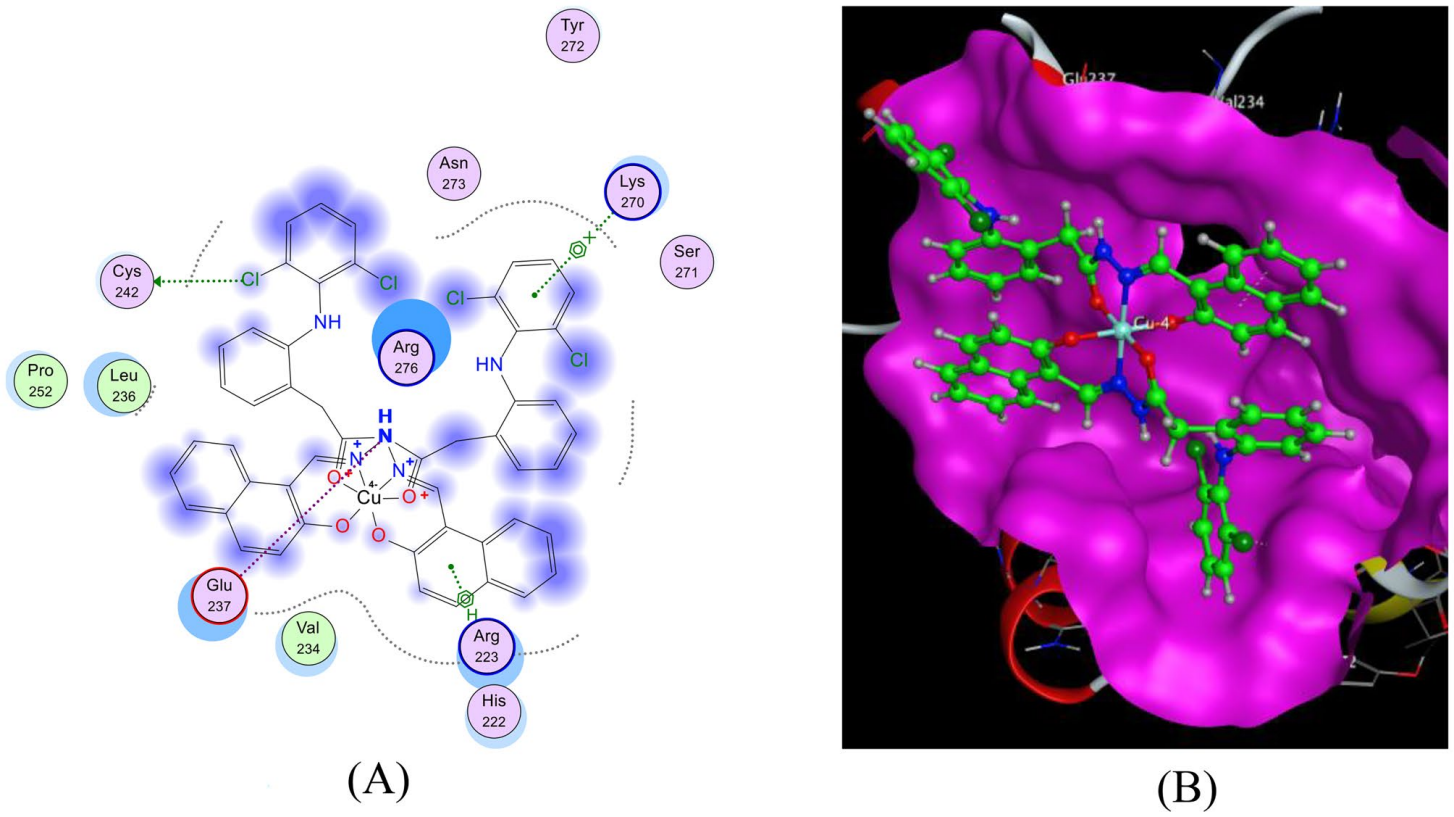
The docking of the Cu(II)-HDN complex with MCF-7:4xo6 receptor: (**A**) 2D and (**B**) 3D view.

The docking analysis with the MCF-7 receptor (Figs. 17 and 18) demonstrated that both the HDN ligand and the Cu(II)-HDN complex formed key interactions with LYS270(A) and CYS242(A), involving hydrogen bonds and π-Cation interactions. The binding energies were −25.29 kcal/mol for HDN and −20.20 kcal/mol for Cu(II)-HDN (Table 8). Despite the HDN ligand having a more favorable binding energy in the docking simulations, the in vitro results revealed that the Cu(II)-HDN complex exhibited greater anticancer activity against MCF-7, possibly due to additional factors in live cell environments, such as metal ion-driven mechanisms.

**Table 8:** The apparent interaction parameters for the HDN ligand and Cu(II)-HDN complex with MCF-7 [PDB code 4xo6].

Receptor	Ligand	Ligand site	Receptor site	Interaction type	Distance (Å)	Energy (kcal/mol)	Rmsd	Binding Energy (kcal/mol)
**MCF-7** (PDB code 4xo6)	HDN	6-ring	N LYS270(A)	Pi-H	3.68	−0.6	1.57	−25.29
		6-ring	Cβ LYS270(A)	Pi-H	3.76	−0.6		
	Cu(II)-HDN	C125	Sγ CYS242(A)	H–D	4.08	−0.9	2.44	−20.20
		N19	Oε1 GLU237(A)	Ionic	3.90	−0.7		
		6-ring	Cδ ARG270(A)	Pi-H	4.04	−0.9		
		6-ring	Nζ LYS270(A)	Pi-cation	3.94	−0.8		

The molecular docking studies of the HDN Schiff base and its metal complexes provided a detailed understanding of their interactions with key biological receptors. While both the HDN ligand and its metal complexes showed strong binding affinities with COX-2, HepG-2, and MCF-7 receptors, the metal complexes—particularly the Cu(II)-HDN complex—demonstrated superior in vitro efficacy. This suggests that metal coordination enhances the biological activity of the HDN ligand, making the metal complexes promising candidates for further research in anticancer and anti-inflammatory drug development.

## Conclusion

In this study, a novel Schiff base, (E)−2-(2-((2,6-dichlorophenyl)amino)phenyl)-N’-((2-hydroxynaphthalen-1-yl)methylene)acetohydrazide, was successfully synthesized and characterized, marking a significant advancement in the development of bioactive compounds. The Schiff base was complexed with various transition and lanthanide metal ions, including Cu(II), Ni(II), Co(II), Gd(III), La(III), and Ag(I), forming well-defined complexes with distinct geometries, such as octahedral, tetrahedral, square antiprismatic, and tricapped trigonal prismatic structures. Comprehensive characterization techniques, including FTIR, UV–vis, ^1^H-NMR, ESR, XRD, and thermal analysis, confirmed these complexes’ successful formation and stability. In vitro studies revealed that these complexes effectively inhibited COX-2, as demonstrated by enzyme-linked immunosorbent assay (ELISA), and exhibited potent cytotoxicity against MCF-7 and HepG-2 cancer cell lines through MTT assays. Moreover, in silico molecular docking studies further confirmed the strong binding interactions of the compounds with COX-2, MCF-7, and HepG-2 receptors, supporting their potential as selective inhibitors with minimal side effects. The synthesized metal complexes showed enhanced biological activity compared to the Schiff base alone, underscoring the critical role of metal coordination in improving drug efficacy. The present results provide a preclinical proof-of-concept for diclofenac-derived Schiff base metal complexes as dual anti-inflammatory and anticancer agents. However, their therapeutic potential remains to be validated through stability, mechanistic, and in vivo investigations.The findings demonstrate the novelty of combining Schiff base chemistry with metal complexation to develop multifunctional compounds that could be used in targeted drug delivery and other biomedical applications.

## Supplementary Information

Supplementary Information.

## Data Availability

We confirm that all data generated or analyzed during this study are included in this published article and its supplementary information files.
