# Supplementary material for: Synthesis, structural elucidation, and molecular docking of diclofenac-derived hydrazone metal complexes with anti-inflammatory and anticancer potential
Source: Sci Rep. 2025 Nov 26;15:42547. doi: 10.1038/s41598-025-27143-1 (PMC12663214; doi:10.1038/s41598-025-27143-1)
Supplement: Supplementary file 1 — Supplementary Information. [file 41598_2025_27143_MOESM1_ESM.docx]

**Synthesis, Structural Elucidation, and Molecular Docking of Diclofenac-Derived Hydrazone Metal Complexes with Anti-Inflammatory and Anticancer Potential**

A.M. Abbas*, H.A. Salemand A. S. Orabi*

*Suez Canal University, Faculty of Science, Chemistry Department, Ismailia-41522, Egypt*

**Corresponding Author:*

[*abbasmamdoh@science.suez.edu.eg* (A.M. Abbas)](mailto:abbasmamdoh@science.suez.edu.eg%20()),[*orabiadel@hotmail.com*](mailto:orabiadel@hotmail.com)(A. S. Orabi)

**Instruments and physical measurements**

Electrical conductivity measurements of solid complexes were carried out at room temperature on freshly prepared 10^‾3^ M DMSO solutions using a WTW digital conductivity meter. Metal content was obtained by using Thermo Fisher Scientific atomic absorption (AA) spectroscopy. The ^1^HNMR spectra of the Schiff base was recorded on LC-MS Triple-quade by ESI ion source, at room temperature using DMSO-*d6* as solvent and tetra- methyl silane (TMS) as an internal standard and chemical shift are given in ppm. Electronic spectra of the metal complexes were recorded on UV-1800- Shmadzu spectrophotometer (double beam spectrophotometer) in the region of 800–200 nm, with quartz cuvette (path length, 1 cm) and studies were performed in DMSO solvent. The Fourier-transform infrared spectroscopy FTIR spectra of the Schiff base and their complexes were performed on a Bruker Tensor 27 spectrophotometer as a KBr discs in the range of 400-4000 cm^-1^. Mass spectrometry was carried out by Shimadzu Qp-2010 plus mass analyzer. Magnetic susceptibility measurements were carried out using the modified Gouy method [1] on MSB-MK1 balance at room temperature using mercury (II) tetrathiocyanatecobaltate(II). The effective magnetic moment, μ_eff_, per metal atom was calculated from the expression μ_eff_ = 2.83B.M., where is the molar susceptibility corrected using Pascal’s constant for the diamagnetism of all atoms in the complexes. Elemental analysis (C, H and N %) were determined by using a Heraus CHN-rapid analyser. Thermal analysis, TGA and DTG were carried out on Shimadzu 60 thermal analyser under a dynamic flow of nitrogen (40 mL/min.) with a linear heating rate of 20˚C/min in the range of 40-800 °C. Electron spin resonance (ESR) of Cu(II) complex was carried out by using BRUKER EMX EPR spectrometer, Germany and using a standard rectangular cavity of ER 4102. X-ray diffraction analysis was carried out by using Bruker D8 DISCOVER XRD, Germany and step size 0.2. X-rays were obtained by applying a potential of 40Kv, a current of 40 mA and source Cu 1.54A.

**Table S1:** ^1^HNMR analysis of the HDN Schiff base (ppm).

| 12.40 (s, 1H, OH), 12.10 (s, 1H, N-NH), 11.60 (s, 1H, -NH-Ar), 10.90 (s,1H, N=CH), 9.23-6.32 (m, 13H, ArH), 3.78 (s, 2H, O=CCH_2_). | **δ (ppm)** |
| --- | --- |
|  | **Ligand**  **Structure** |

**Table S2:** Molecular ion peaks and their *m/z* values (%) for the diclofenac hydrazide and HDN Schiff base.

| **Molecular ion peak** | ***m/z* (%)** | **Compound** |
| --- | --- | --- |
| 310.85(2) | 310.85(2), 309.85(1), 242.85(6), 214.90(37), 179.95(14), 151.00(15), 107.05(4), 89.10(12), 77.00(11), 63.00(7), 51.00(8). | **Diclofenac hydrazide** |
| 464.65 (1) | 464.65 (1), 462.65 (1), 422.90 (0.3), 367.95 (1), 338.95 (1), 313.00 (1), 277.75 (27), 213.80 (67), 185.90 (100), 168.90 (33), 128.00 (19), 115.00 (23), 89.00 (9), 77.00 (13), 55.00 (6). | **Schiff base (HDN)** |

**Table S3**: TGA and DTG analysis of the prepared compounds.

| **Compound** | **Temp.**  **range(^o^C)** | **DTG**  **temp. (^o^C)** | **Mass loss %** | | **Process** | **Expected prodcts** | **Residue** |
| --- | --- | --- | --- | --- | --- | --- | --- |
|  |  |  | **Found** | **Calcd.** |  |  | **Found**  **(Calcd.)%** |
| Ligand (HDN) | 227-435  435-652 | 237,292  545,612,639 | 66.1  31.04 | 67.30  30.30 | Ligand decomp.  Final decomposition | 0.67 HDN  0.31 HDN | --- |
| [Co(DN)_2_] | 23-339  339-469  469-751 | 317  387,444  512 | 28.03  26.48  37.71 | 28.08  26.38  38.63 | 1^st^ Ligand decomp.  2^nd^ Ligand decomp.  Final decomposition | 0.30 HDN  0.28 HDN  0.40 HDN | CoO  7.78  (7.60) |
| [Ni(DN)_2_] | 38-361  361-395  395-439  439-689 | 327  387  418  483 | 25.25  18.96  12.33  34.02 | 25.39  18.85  12.70  33.93 | 1^st^ Ligand decomp.  2^nd^ Ligand decomp.  3^rd^ Ligand decomp  Final decomposition | 0.27 HDN  0.20 HDN  0.14 HDN  0.36 HDN | NiO  9.44  (7.58) |
| [Cu(DN)_2_]2.5H_2_O | 23-166  166-336  336-707 | 42  293  486 | 4.65  19.59  67.63 | 4.35  19.73  67.28 | Water of crystallization  Ligand decomp.  Final decomposition | 2.5H_2_O  0.22 HDN  0.75 HDN | CuO  8.13  (7.68) |
| [Gd(HDN)_2_(NO_3_)_2_]NO_3_.4H_2_O | 32-115  115-377  377-648 | 52  202,277  508 | 5.13  30.71  50.85 | 5.36  30.71  50.81 | Water of crystallization  NO_3_ liberation +Ligand decomp.  Final decomposition | 4H_2_O  3HNO_3_ + 0.25 HDN  0.74 HDN | Gd  13.31  (11.70) |
| [La(HDN)(NO_3_)_2_(H_2_O)_4_]NO_3_ | 131-436  436-666 | 180,278  509 | 30.58  52.68 | 30.58  52.83 | Coordination sphere + NO_3_ liberation  Ligand decomp. | 4H_2_O + 3HNO_3_  0.98 HDN | La  16.74  (16.13) |
| [Ag(DN)(H_2_O)] | 29-416  416-693 | 275,363  574 | 16.48  62.90 | 16.48  63.05 | Coordination sphere + Ligand decomp.  Final decomposition | H_2_O + 0.17 HDN  0.80 L | Ag  20.62  (18.31) |

**Table S4:** The number of spins (spin concentration)of the Cu(II)-HDN complex.

| **Cu(II)-HDN complex** | **Value** |
| --- | --- |
| **H_0_** | **3337.10** |
| **∆H** | **81.70** |
| **A** | **965275.60** |
| **K** | **1.00*10^13^** |
| **H_m_** | **8.00** |
| **G_e_** | **4480.00** |
| **√P** | **1.41** |
| **N_s_** | **2.127*10^21^** |
| **g\|\|** | **2.06964** |
| **g┴** | **2.10154** |

**Table S5:** X-ray diffraction results for the Cu(II)-HDN complex.

| **FWHM**  **[^o^2**𝜽] | | \| ** **  **(nm)** \| \| --- \| | | 𝛅 | |
| --- | --- | --- | --- | --- | --- | --- |
| 0.141^°^ | | 59.7 | | 2.81×10^-4^ | |
| 0.170 | | 48.5 | | 4.25×10^-4^ | |
| 0.464^°^ | | 17.2 | | 3.38×10^-3^ | |
| 0.539^°^ | | 15.0 | | 4.44×10^-3^ | |
| 0.596^°^ | | 13.6 | | 5.41×10^-3^ | |
| 0.312^°^ | | 25.9 | | 1.49×10^-3^ | |
| **Average** | **29.98** | |  | |  |

**Table S6:** The crystallographic parameters computed for the Cu(II)-HDN complex.

| \| **Parameters** \| \| --- \| | **Cu(II)-HDN** |
| --- | --- | --- |
| Molecular Formula | [Cu(DN)_2_]2.5H_2_O |
| Molecular weight | 1035.26 |
| Crystal system | Triclinic |
| Space group | P1 |
| Unit cell dimension | |
| a (Å) | 18.22 |
| b (Å) | 11.99 |
| c (Å) | 8.59 |
| α (^o^) | 99.09 |
| β (^o^) | 95.89 |
| γ ^^ | 72.41 |
| Volume (Å^3^) | 1763.18 |
| Volume per atom (Å^3^) | 27.46 |
| Z | 2 |

**Table S7:** Inhibition of COX-1 and COX-2 *in vitro* (**IC_50_**).

| **Cpd.** | **COX-1**  **IC_50_ (µM)** | **COX-2**  **IC_50_ (µM)** | **COX-2 S.I.^*^**  **IC_50_ (µM)** |
| --- | --- | --- | --- |
| **Ligand [HDN]** | **10.47** | **0.06** | **174.50** |
| **Co(II)-HDN** | **8.50** | **0.62** | **13.71** |
| **Gd(III)-HDN** | **6.00** | **0.95** | **6.31** |
| **Ag(I)-HDN** | **7.50** | **0.53** | **14.15** |
| **Diclofenac sodium** | **3.80** | **0.84** | **4.52** |
| **Rofecoxib** | **14.50** | **0.02** | **725.00** |
| **Indomethacin** | **0.10** | **0.08** | **1.25** |
| **Celecoxib** | **14.50** | **0.05** | **290.00** |

^*^ selectivity index (COX-1 IC_50_/COX-2 IC_50_).

**Table S8**: The HDN ligand's *in vitro* inhibition percentages and IC_50_ values against HepG-2 and MCF-7 cell lines.

| **Cpd.** | **Conc.**  **µg/ml** | **Inhibition %** | **IC_50_** | |
| --- | --- | --- | --- | --- |
| **MCF-7** | --- | 0.00 | **µg/ml** | **µM** |
| **Ligand (HDN)** | 1000 | 97.49 | **82.61** | **177.90** |
|  | 500 | 97.32 |  |  |
|  | 250 | 89.17 |  |  |
|  | 125 | 71.36 |  |  |
|  | 62.5 | 52.54 |  |  |
|  | 31.25 | 7.98 |  |  |
| **HepG-2** | --- | 0.00 | --- | --- |
| **Ligand (HDN)** | 1000 | 97.00 | **70.49** | **151.80** |
|  | 500 | 97.24 |  |  |
|  | 250 | 96.88 |  |  |
|  | 125 | 80.42 |  |  |
|  | 62.5 | 61.43 |  |  |
|  | 31.25 | 12.93 |  |  |

**Table S9**: The *in vitro* inhibition % and IC_50_ of the prepared complexes against MCF-7 and HepG-2 cell lines.

| **Cpd.** | **Conc. µM/ml** | **Inhibition %** | **IC_50_** |
| --- | --- | --- | --- |
| **MCF-7** | -------- | 0 | **µM** |
| **Gd(III)-HDN** | 10.00 | 96.86 | **0.80** |
|  | 5.00 | 96.71 |  |
|  | 2.50 | 75.34 |  |
|  | 1.25 | 70.06 |  |
|  | 0.62 | 50.83 |  |
|  | 0.31 | 18.33 |  |
| **Cu(II)-HDN** | 10.00 | 97.49 | **0.65** |
|  | 5.00 | 97.39 |  |
|  | 2.50 | 97.18 |  |
|  | 1.25 | 84.90 |  |
|  | 0.62 | 63.27 |  |
|  | 0.31 | 16.98 |  |
| **HepG-2** | --- | 0.00 | --- |
| **Gd(III)-HDN** | 10.00 | 95.27 | **2.47** |
|  | 5.00 | 81.14 |  |
|  | 2.50 | 53.05 |  |
|  | 1.25 | 7.98 |  |
|  | 0.62 | 0.40 |  |
|  | 0.31 | 0.45 |  |
| **Cu(II)-HDN** | 10.00 | 97.38 | **1.00** |
|  | 5.00 | 97.32 |  |
|  | 2.50 | 85.53 |  |
|  | 1.25 | 61.65 |  |
|  | 0.62 | 35.16 |  |
|  | 0.31 | 2.96 |  |

**
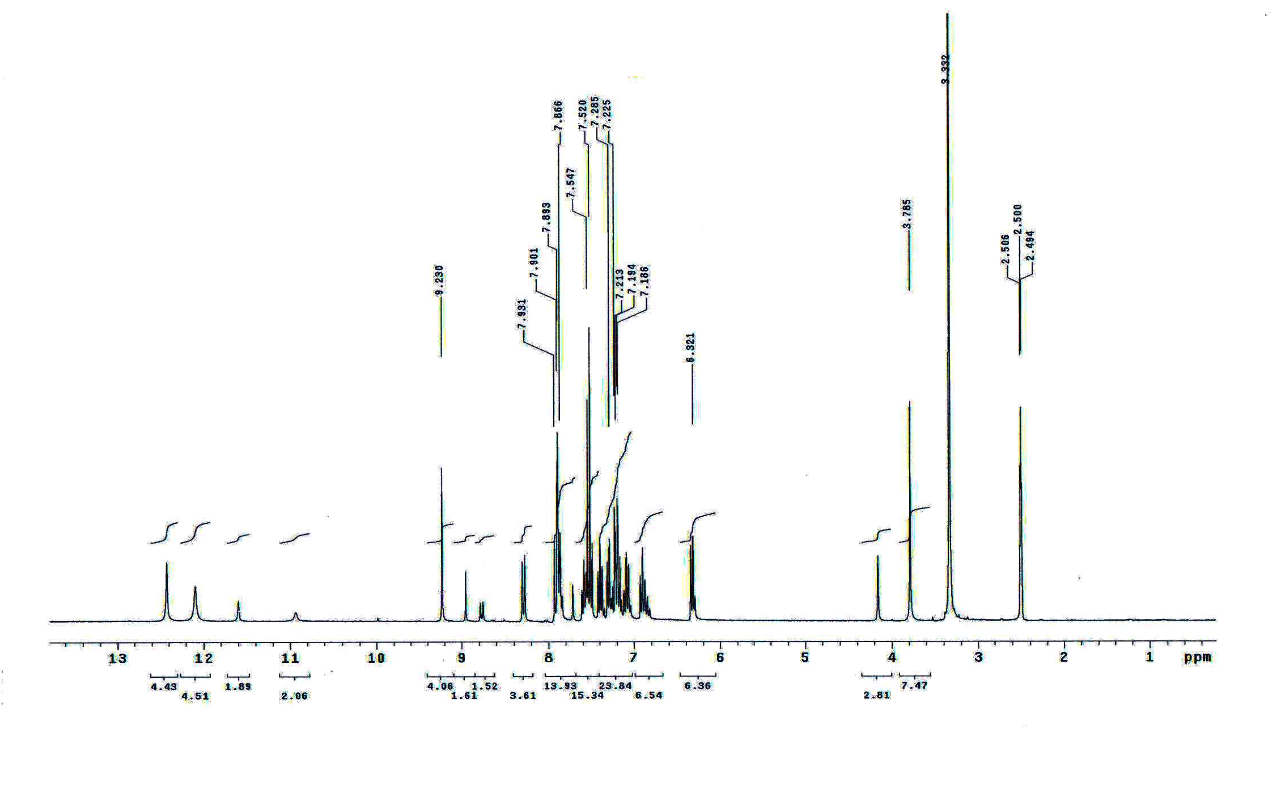
**

**Figure S1:** ^1^HNMR spectrum of the HDN ligand.


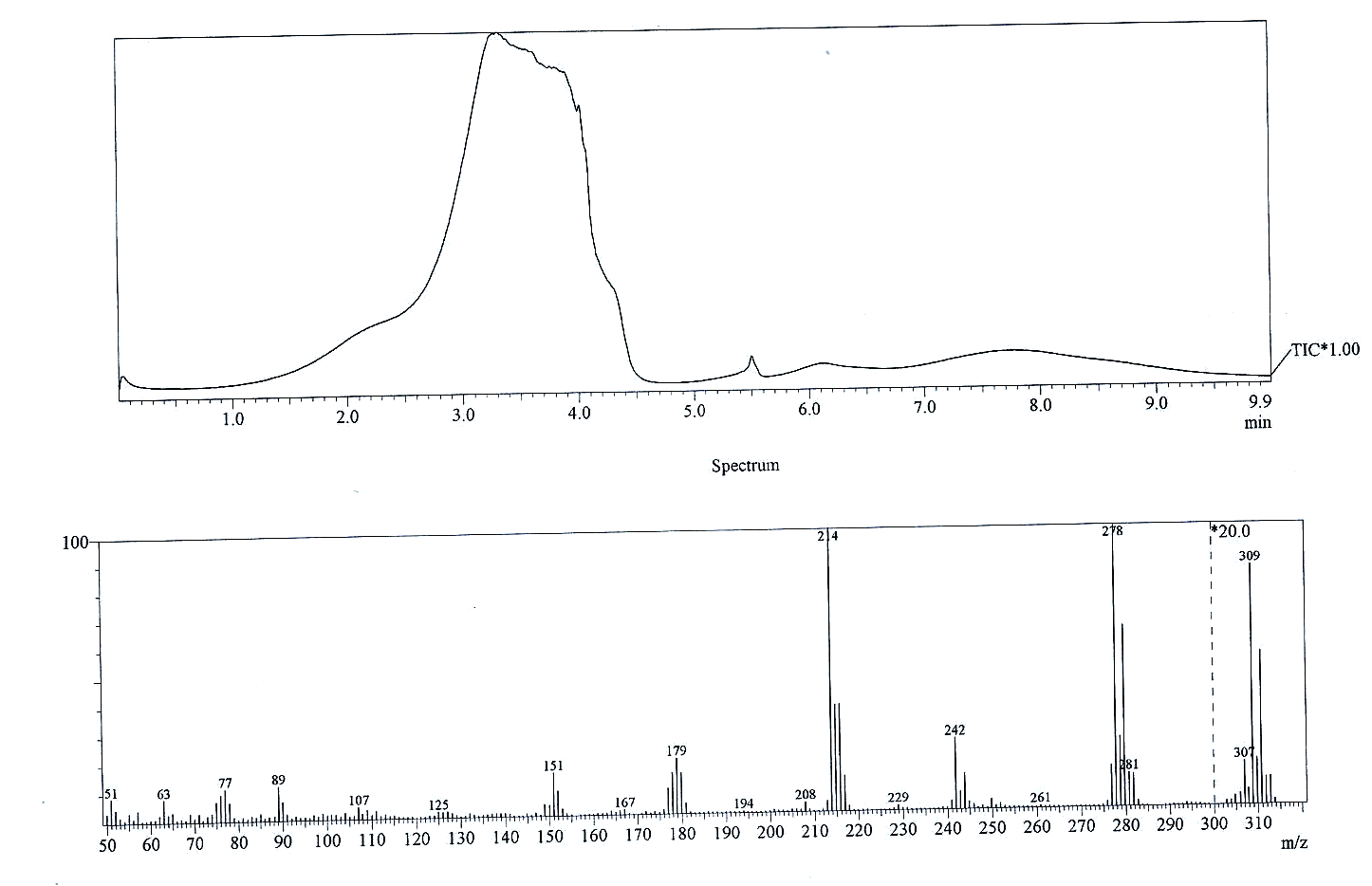


**Figure S2:** Chromatogram and mass spectrum of the diclofenac hydrazide.


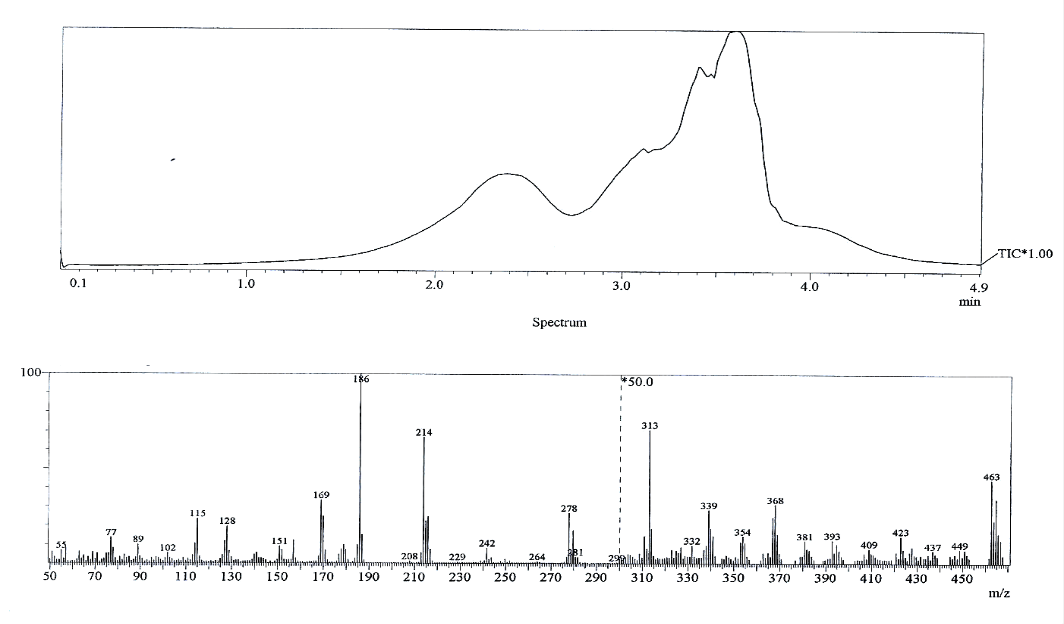


**Figure S3:** Chromatogram and mass spectrum of the HDN ligand.

**Figure S4:** FTIR spectrum of the diclofenac acid.  **Figure S5:** FTIR spectrum of the diclofenac ethyl.

**Figure S6:** FTIR spectrum of the diclofenac hydrazide.  **Figure S7:** FTIR spectrum of the HDN ligand.

**Figure S8:** FTIR spectrum of the Co(II) complex.  **Figure S9:** FTIR spectrum of the Ni(II) complex.

**Figure S10:** FTIR spectrum of the Cu(II) complex.  **Figure S11:** FTIR spectrum of the Gd(III) complex.

**Figure S12:** FTIR spectrum of the La(III) complex.  **Figure S13:** FTIR spectrum of the Ag(I) complex.

**Figure S14:** UV-Vis. spectrum of the HDN Schiff base.

**S15:** UV-Vis. spectrum of [Co(DN)_2_]. **S16:** UV-Vis. spectrum of [Ni(DN)_2_].

**S17:** UV-Vis. spectrum of [Cu(DN)_2_]2.5H_2_O.  **S18:** UV-Vis. spectrum of [Gd(HDN)_2_(NO_3_)_2_]NO_3_.4H_2_O.

**S19:** UV-Vis. spectrum of [La(HDN)(NO_3_)_2_(H_2_O)_4_]NO_3_. **S20:** UV-Vis. spectrum of [Ag(DN)(H_2_O)].

**Figure S21:** The thermal decomposition of the **Figure S22:** The thermal decomposition of the

[Co(DN)_2_] complex. [Ni(DN)_2_] complex.

**Figure S23:** The thermal decomposition of the **Figure S24:** The thermal decomposition of the

[Cu(DN)_2_]2.5H_2_O complex. [Gd(HDN)_2_(NO_3_)_2_]NO_3_.4H_2_O complex.

**Figure S25:** The thermal decomposition of the **Figure S26:** The thermal decomposition of the. [La(HDN)(NO_3_)_2_(H_2_O)_4_]NO_3_ complex [Ag(DN)(H_2_O)] complex.

- - 1. **Electron spin resonance (ESR) measurement**

*The number of spins (spin concentration) was calculated by using formula:

N_s_ = k . [H_o_ (∆H)^2^ A / 2] / [H_m_ G_e_ √P]

Where:

- k is a factor based on the EPR spectrometer's experimental conditions, equal to 10^13^.

- Ho represents the magnetic field at the resonance peak in gauss.

- (∆H) is the peak width.

- A stands for the EPR signal intensity (EPR signal peak height divided by sample weight).

- Hm is the modulation amplitude value.

- √P denotes the square root of microwave power in mW.

- Ge represents receiver gain.

**
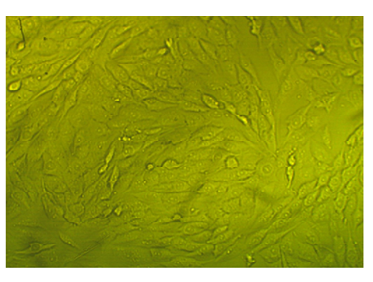

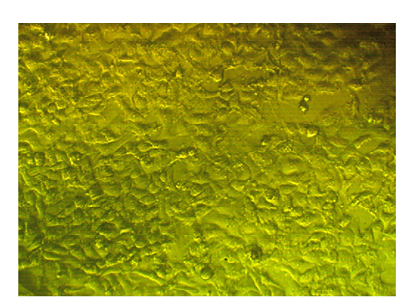
**

**S21:** Control HepG-2 cells, **S22:** Control MCF-7 cells,

Organism: homo sapiens, human. Organism: homo sapiens, human

Tissue: liver Tissue: mammary gland, breast; derived from metastatic site:

Cell type: epithelial pleural effusion

Culture properties: adherent Cell type: epithelial

Diseases: hepatocellular carcinoma Culture properties: adherent

Diseases: adenocarcinoma

**
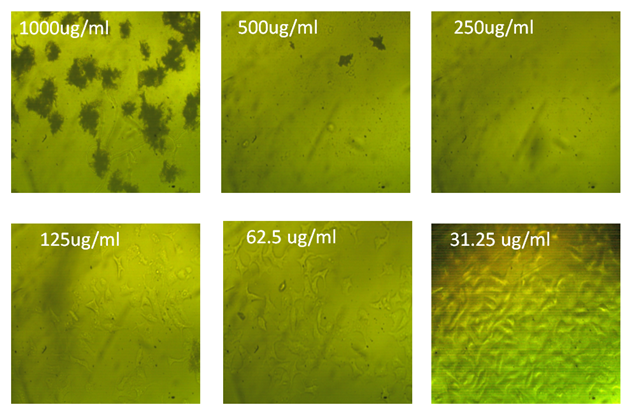

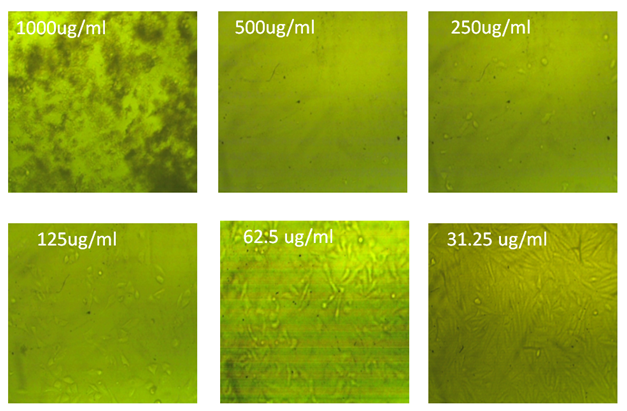
**

**S23:** Effect of the prepared ligand on **S24:** Effect of the prepared ligand on

HepG-2 cells at different concentrations.MCF-7 cells at different concentrations.

**
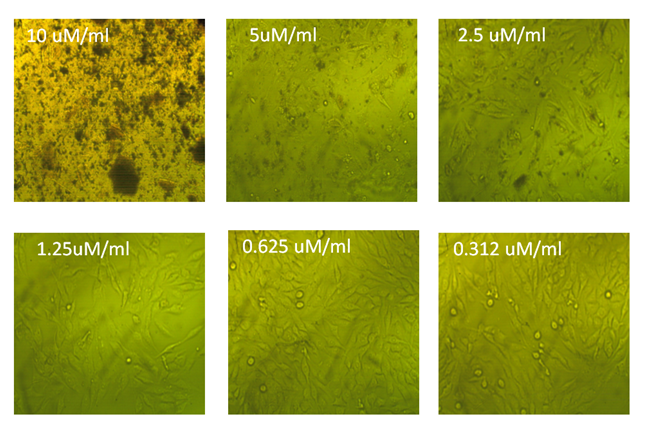

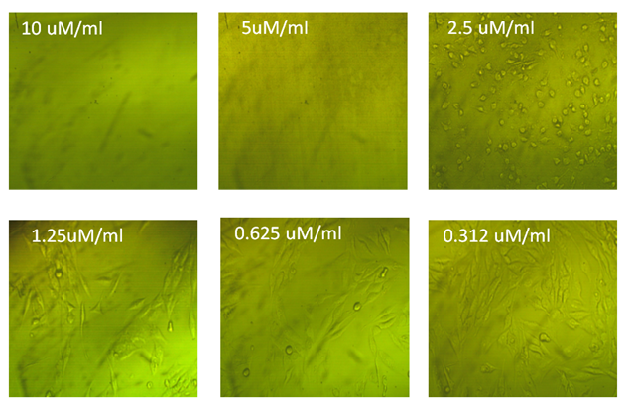
**

**S25:** Effect of the Gd(III) complex on **S26:** Effect of the Cu(II) complex on

HepG-2 cells at different concentrations. HepG-2 cells at different concentrations.

**
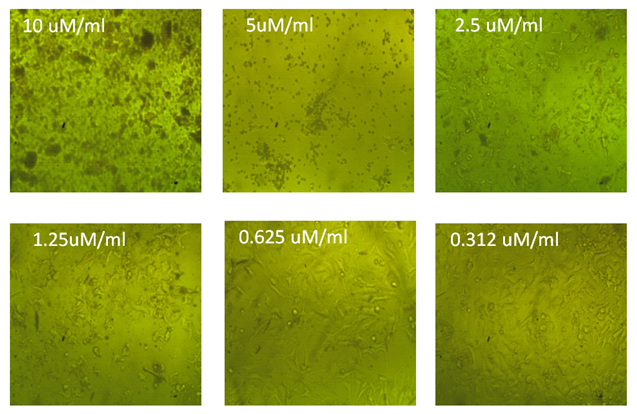

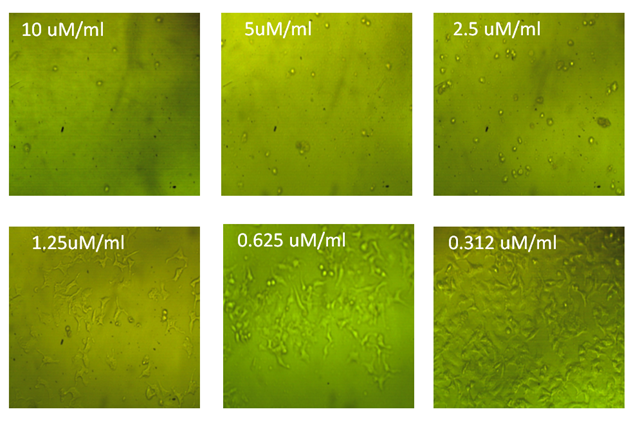
**

**S27:** Effect of the Gd(III) complex on **S28:** Effect of the Cu(II) complex on

MCF-7 cells at different concentrations. MCF-7 cells at different concentrations.


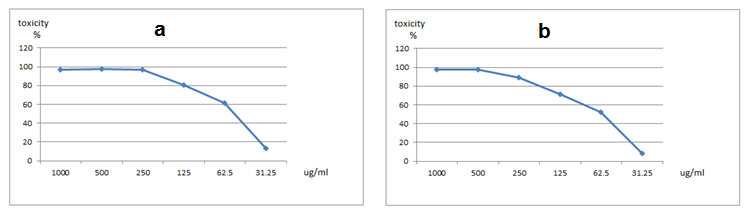


**S29:** Effect of the prepared ligand on a) MCF-7 and b) HepG-2 cancer cells at different conc.

**
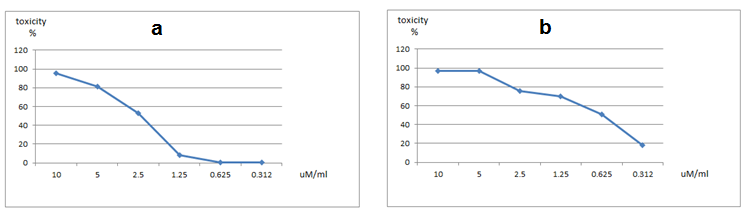
**

**S30:** Effect of the Gd(III) complex on a) MCF-7 and b) HepG-2 cancer cells at different conc.

**
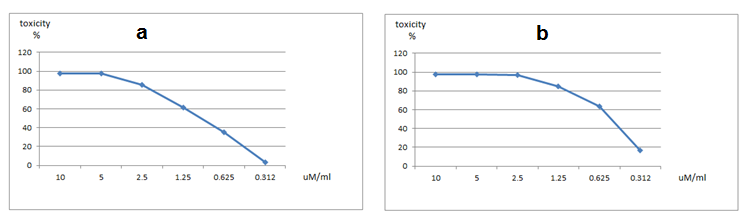
**

**S31:** Effect of the Cu(II) complex on a) MCF-7 and b) HepG-2 cancer cells at different conc.

[1] B. Figgis, In J. Lewis & RG Wilkins (Eds.), Modern coordination chemistry: Principles and methods, New York: Wiley, 1960.
